# Suicide rates before and during the COVID-19 pandemic: a systematic review and meta-analysis

**DOI:** 10.1007/s00127-024-02617-1

**Published:** 2024-02-14

**Authors:** Ana Paula da Cunha Varella, Eve Griffin, Ali Khashan, Zubair Kabir

**Affiliations:** 1 School of Public Health-UCC, University College Cork, Western Gateway Building (4th Floor), Cork City, co., Cork, Ireland; 2 National Suicide Research Foundation, Cork, Ireland

**Keywords:** Suicide, COVID-19, Mental health, Pandemic

## Abstract

**Purpose:**

The effects of the COVID-19 pandemic on mental health issues such as depression and anxiety are well-documented in the literature, but its influence on suicidal patterns shows divergent results. We aim to comprehensively synthesize evidence on potential changes or stability of suicide rates during the COVID-19 pandemic worldwide.

**Methods:**

A comprehensive search of studies reporting suicide rates before and during the COVID-19 pandemic was conducted. Eligible studies reported incidences of confirmed suicides (suspected in special cases) for two time periods, before (until February 2020) and during (from March 2020 to June 2021) the COVID-19 pandemic. A meta-analysis of proportions using a random-effect model was performed to estimate the pre- and during-pandemic incidence rates of suicide with 95% confidence intervals. Differences in pre- and during-pandemic rates were formally tested using a heterogeneity test.

**Results:**

A total of 34 studies were included in the review capturing suicide data from over 40 countries and regions. The meta-analysis outputs did not indicate a significant change in suicide rates during the COVID-19 pandemic. The pooled suicide rate in the studied period before the pandemic was 11.38 per 100,000 (95% CI 9.35–13.42) and in the period during the pandemic was 10.65 per 100,000 (95% CI 8.61–12.68).

**Conclusion:**

No significant change in suicide rates was observed during the COVID pandemic from a global perspective for the periods examined. A longer follow-up can provide additional insights into such suicide trends globally. Improvements in data reporting, specifically with implementation of real-time surveillance, is imperative to provide adequate suicide prevention and support.

**Supplementary Information:**

The online version contains supplementary material available at 10.1007/s00127-024-02617-1.

## Introduction

Since the beginning of the COVID-19 outbreak, officially declared a pandemic by the World Health Organization on March 11, 2020 [1], almost 7 million people have lost their lives as a result of the virus [2]. In an attempt to control the spread of the SARS-CoV-2 virus, most countries adopted measures of movement restriction and personal isolation. These periods of restriction and lockdown were associated with impacts on several sectors including economic activity [3, 4], the health system [5–8], and education [9]. There is evidence that while restrictions at a national level were effective in reducing the spread of the virus and levels of serious illness, studies have also shown that these restrictions negatively impacted the mental health of the general population [10]. Several studies internationally have demonstrated a high prevalence of depression [11–13], anxiety [13], post-traumatic stress disorder [11, 12], and insomnia [12, 13] during the pandemic among the general population [11, 12], healthcare workers [12–14], and patients with COVID-19 [12, 13].

While it is clear that the COVID-19 pandemic has had a significant negative impact on mental health, less is known about its impact on rates of suicide internationally. Suicide is a complex public health problem, and several factors can influence an individual’s risk of suicide, including biological, clinical, psychological, social, cultural, and environmental factors [15]. At a population level, suicide rates have been known to rise during periods of economic crisis [16], during and after natural disasters [17], and during and after post-war periods [18–20]. Previous disease-related public health emergencies, including the Severe Acute Respiratory Syndrome (SARS) outbreak in 2003, the Great Influenza Epidemic more than 100 years ago and the Russian Influenza at the end of the nineteenth century [21], have been shown to be associated with increased rates of suicide.

Although research estimating the prevalence of suicide during the COVID-19 pandemic is documented, the findings are mixed. Much research comes from high-income countries, which showed no evidence of an increase in suicide rates in the early months of the pandemic [22]. Published studies vary in their methodology, the use of pre-pandemic trends, and an exploration of specific regions (e.g., low–middle-income countries) and groups (age and sex differences) [23]. Systematically reviewing and synthesizing data on rates of suicide globally during the COVID-19 pandemic can provide clearer picture of the patterns of suicide during the pandemic and an additional insight into informing public and mental health measures for future public health emergencies and pandemics. Our research remains relevant despite the recent publication of a similar study, which was published after we completed our research. [24] First, our systematic review included a larger number of papers, offering a more comprehensive analysis of the literature. Second, our research encompassed data from a wider range of countries/locations, providing a more diverse perspective on the impact of the COVID-19 pandemic on suicide rates. Lastly, we conducted a thorough review of suicide data sources, considering their quality, which enhances the reliability and validity of our findings.

## Methods

The method is described in accordance with the Preferred Reporting Items for Systematic Reviews and Meta-Analyses guidelines (PRISMA) [25]. The methods were pre-specified in a protocol that was registered with the PROSPERO International Prospective Register of Systematic Reviews (CRD42022342011).

### Eligibility criteria

We included studies reporting suicide rates or proportions as outcome both during and within 10 years prior to COVID-19 pandemic, published in peer-reviewed journals, reporting confirmed or suspected suicides (high-quality studies only). Exclusion criteria included studies reporting other suicide behavior, or suspected suicides (if of inferior quality) as well as if the data sources or data of one period only was missing. Grey literature, abstracts, reviews, preprints, and non-English studies were not included. The eligibility criteria changed from our PROSPERO protocol, which initially included suicide cases within 3 years before the pandemic as the comparison group, but was expanded to 10 years to avoid excluding potentially relevant studies based solely on the length of the comparison period.

### Search strategy

A literature search of published studies on four databases was conducted without date restrictions, with potentially eligible studies published until 1st June 2022 searched. A combination of keywords and MeSH was used for the search, with “COVID-19” and “SARS-CoV-2” used as exposure terms and “suicid*” as the outcome term. The four comprehensive databases: PubMed, Web of Science, Scopus, and PsycINFO, were searched. The reference list of included studies was also manually searched. The search settings for each database are provided in Online Resource 1.

### Study selection

All retrieved studies were added to Zotero software and duplicates were removed. Two authors (AV and EG) then independently screened the titles and abstracts of all papers, excluding any that did not meet the eligibility criteria. Eligible studies were reviewed for papers where the title and abstract did not provide enough information to decide inclusion or exclusion. Reasons for exclusion during the full-text review were recorded in a detailed table available in Online Resource 2.

### Data extraction

Two authors (AV and EG) used a standardized form to independently extract data from the included studies. Data retrieved included author, year of publication, country/region/city, population, definition of suicide, pre-pandemic, and during-pandemic time periods, sources of data, frequency of data reporting, suicide rate, and data on subgroups such as gender, age, and ethnicity. Data were extracted from all selected studies fully, including multiple frequencies of data report if provided.

### Quality appraisal

The Joanna Briggs Institute Critical Appraisal Tool for Studies Reporting Prevalence Data (JBI) [26] was used to assess the quality of the included studies. This tool has nine questions and evaluates aspects such as sampling, statistical analysis methods, and standardization. Questions related to sampling and response rate were not considered relevant and marked as “not applicable” for all studies. If the study did not explain how the suicide diagnosis was made, questions 6 and 7, which evaluate the method of identification of the condition, were marked as “unclear”. Two authors (AV and EG) independently performed the quality appraisal process. The summary of the quality appraisal is available in Online Resource 3.

### Statistical analysis

All included studies were summarized descriptively, according to the following categories: author, year of publication, place, population, definition of suicide, definition of pre- and during-pandemic periods, frequency of data report, data source (suicide/population) and if the study report data by gender and age groups.

Pooled estimates of proportions for respective time periods were performed using the Metaprop [27] command in Stata. As high heterogeneity in the pooled incidence rates was noted, a random-effect model was used. The pooled incidence of suicide with 95% confidence interval for the pre- and during-pandemic periods was calculated separately for women, men, and the general population. All suicide rates (crude and age-specific) presented in this study are per 100,000 persons. If not provided by the study, population data used to calculate the pooled suicide rate were sourced from official government statistical websites. Population data provided by studies which did not specify the data source were not used. Official governmental statistical data were searched and used instead. If more than one study provided data from the same area, data from only one study were included in the statistical analysis. The studies of longer time periods were prioritized for inclusion. If one study provided data of more than one country/region/area, each location was analyzed separately. To calculate the pooled suicide rates, a 12-month period mean or annual data were used. Methods to calculate the 12-month period mean data are explained in Online Resource 4.

For studies which did not state a specific pre- and during-pandemic periods, April 2020 was defined as the beginning of the pandemic period. This was based on a recent study in the Lancet [22]. The original pre- and during-pandemic periods, as reported in studies, were unaltered for analysis. Differences in pre- and during-pandemic rates were formally tested using a heterogeneity test, with significance determined by both 95% confidence intervals and *p* value, with *p* < 0.05 assumed to be significant. A priori subgroup analysis, such as by WHO regions and gender, was undertaken. Suicide data by age, ethnicity, and socio-demographic index (were available) were described narratively, because of paucity of data.

Publication bias was assessed using a funnel plot. The Grading of Recommendations Assessment, Development, and Evaluation (GRADE) [28] approach was used to rate the certainty of findings.

To explore heterogeneity further, we performed a post hoc meta-regression analysis. In accordance with the recommendations outlined in the Cochrane Handbook, we restricted the meta-regression to comparison groups that included a minimum of ten studies. [29]

## Results

**Fig. 1:**
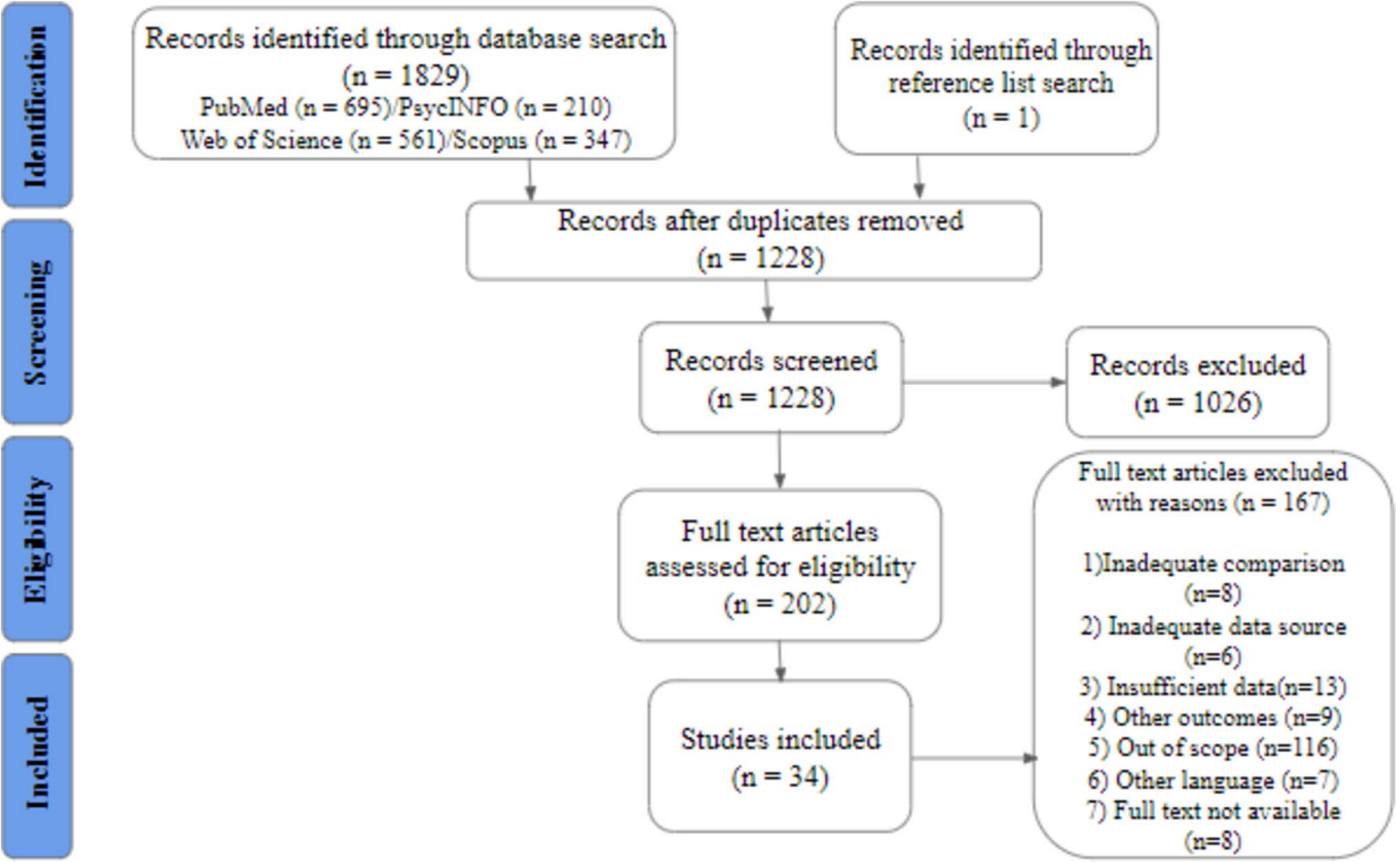
A total of 1,829 records were identified through database search and 1 through reference list search. After deduplication, 1,228 records were initially screened, resulting in 1,026 exclusions. A total of 202 full texts were assessed for eligibility, which resulted in the inclusion of 34 studies. The reasons for exclusion are inadequate comparison, inadequate data source, insufficient data, other outcome, out of scope, other language, and full text not available

A total of 1,829 articles were identified by the initial search of the databases. The study selection process resulted in 34 included studies. The details of the process are shown by the PRISMA flow diagram (Fig. 1).

### Characteristics of the included studies

A total of 34 studies were included from 25 countries across 4 of the 6 WHO regions. The outcome measurement varied across the studies, with most defining the outcome as “confirmed suicide cases.” The great majority of studies (94%) reported data on the general population. Of the included studies, 47% presented data by gender and 29% presented data by age groups. Two studies [30, 31], both from the United States, presented data by ethnic group. One study, from India [32], reported suicide rates by socio-demographic index.

The majority of studies defined the pre-pandemic period as between 2017 and 2019, and considered 2020 (fully or partially) as the during-pandemic period. Some studies presented data for 2021. In addition, the studies were divided into different categories regarding the frequency of data report, including annual, monthly, and other frequencies.

Data sources of the studies varied in terms of the type of suicide data reported and included real time surveillance (e.g., interim Queensland Suicide Register; *n* = 4), governmental records (e.g., Japanese Ministry of Health, Labor and Welfare and Norwegian Cause of Death Registry; *n* = 20), police records (e.g., Catalonia Regional Police Department; *n* = 3), and coroners/chief examiner’s office (e.g., BC Coroners Service; *n* = 7). Only 44% of the studies specified the data source of population data used to calculate the suicide rates, although not all of them provided the actual population data. A summary of all the included studies and their characteristics is shown in Online Resource 5.

### Meta-analysis

In total, data extracted from 21 studies, representing a total of 34 countries or regions, were included in the meta-analysis. The reasons for excluding studies and datasets are explained in Online Resource 6. The pooled incidence of suicide for the pre-COVID-19 pandemic period (until February 2020) was estimated at 11.38 per 100,000 (95% CI 9.35–13.42), and 10.65 per 100,000 (95% CI 8.61–12.68) during the COVID-19 pandemic (from March 2020 to June 2021). Heterogeneity tests between the two time periods showed no significant difference in the suicide rates in both periods (*p* = 0.61, CI pre- and during-pandemic CIs 9.35–13.42 and 8.61–12.68; See Table 1).

**Table 1:** Estimated pre- and during-pandemic periods suicide rates/100,000 by study/area

	Pre-pandemic period (until February 2020)	During-pandemic period (from March 2020 to June 2021)
Study (location)	Suicide rate (95% CI)	Suicide rate (95% CI)
Japan	16.37 (16.15–16.59)	16.71 (16.49–16.94)
South Korea	26.57 (26.13–27.02)	24.74 (24.31–25.17)
Taiwan	16.39 (15.88–16.91)	15.49 (14.99–16)
Guangdong, China	3.79 (3.68–3.91)	3.13 (3.02–3.23)
New Zealand	13.08 (12.12–14.1)	11.5 (10.61–12.47)
New South Wales, Australia	11.6 (10.88–12.36)	10.77 (10.09–11.51)
Queensland, Australia	14.43 (13.43–15.5)	14.67 (13.66–15.75)
Victoria, Australia	10.72 (9.96–11.53)	9.89 (9.16–10.67)
Queensland, Victoria and Tasmania, Australia	12.74 (12.13–13.38)	12.61 (12–13.25)
Thailand	3.79 (3.64–3.94)	6.08 (5.89–6.27)
Nepal	18.86 (18.37–19.37)	25.23 (24.66–25.81)
New Delhi, India	10.93 (10.05–11.88)	7.76 (7.03–8.57)
Croatia	15.58 (14.39–16.87)	14.72 (13.57–15.98)
Poland	13.52 (13.16–13.9)	14.26 (13.89–14.64)
Romania	11.4 (9.16–14.18)	9.44 (7.44–11.99)
Norway	11.87 (10.99–12.83)	11.85 (10.97–12.81)
Emilia-Romagna, Italy	8.1 (7.29–9.00)	8.6 (7.78–9.51)
Milan and Monza, Italy	3.48 (2.96–4.1)	2.34 (1.92–2.86)
Tyrol, Austria	17 (14.3–20.21)	11.88 (9.67–14.6)
Rhineland-Palatinate, Germany	11.00 (10.03–12.07)	11.00 (10.03–12.06)
Catalonia, Spain	6.97 (6.41–7.58)	7.15 (6.58–7.77)
Ten regions in England	9.64 (9.12–10.19)	11.32 (10.75–11.91)
The Netherlands	10.19 (9.73–10.67)	10.06 (9.6–10.54)
British Columbia, Canada	12.62 (11.68–13.64)	11.36 (10.47–12.32)
Alberta, Canada	16.78 (15.51–18.15)	11.29 (10.26–12.42)
Nova Scotia, Canada	13.52 (11.37–16.07)	11.3 (9.39–13.61)
Canada	10.82 (10.49–11.16)	7.34 (7.07–7.62)
California, USA	11.24 (10.92–11.58)	9.12 (8.83–9.43)
Cook County, USA	9.25 (8.46–10.12)	8.75 (7.98–9.59)
New Jersey, USA	8.14 (7.58–8.74)	6.81 (6.3–7.36)
Maryland, USA	8.84 (8.13–9.62)	9.13 (8.41–9.92)
Chile	10.63 (10.16–11.13)	8.4 (7.98–8.84)
Mexico City, Mexico	5.92 (5.45–6.44)	5.68 (5.21–6.18)
Peru	1.81 (1.67–1.97)	1.70 (1.56–1.85)
Pooled suicide rate	11.38 (9.35–13.42)	10.65 (8.61–12.68)
Heterogeneity test	(*I*^2^ = 99.89%, *p* < 0.01)	(*I*^2^ = 99.89%, *p* < 0.01)

### Sub-group analysis

Heterogeneity tests between the two time periods for the four WHO regions showed no significant difference in the suicide rates (*p* = 0.88, *p* = 0.83, *p* = 0.77 and *p* = 0.99, respectively); (See Table 2). The forest plots of the pooled suicide rate for both periods by region are exhibited in Online Resource 7.

**Table 2:** Estimated pre- and during-pandemic suicide rates/100,000 by WHO region

WHO region	Pre-pandemic suicide rate (95% CI) (until February 2020)	During-pandemic suicide rate (95% CI) (from March 2020 to June 2021)
Western Pacific	13.96 (7.93–20.00) Heterogeneity (*I*^2^ = 99.96%, *p* < 0.01)	13.28 (CI 7.09–19.47) Heterogeneity (*I*^2^ = 99.96%, *p* < 0.01)
South East Asia	11.19 (0.32–22.06) Heterogeneity (*I*^2^ = –, *p* = 0.04)	13.02 (0.66–25.39) Heterogeneity (*I*^2^ = –, *p* = 0.04)
Europe	10.70 (8.52–12.88) Heterogeneity (*I*^2^ = 99.08%, *p* < 0.01)	10.23 (CI 7.56–12.89) Heterogeneity (*I*^2^ = 99.42%, *p* < 0.01)
Americas	9.94 (6.67–13.12) Heterogeneity (*I*^2^ = 99.82%, *p* < 0.01)	8.23 (CI 5.85–10.61) Heterogeneity (*I*^2^ = 99.74%, *p* < 0.01)

Suicide data from nine studies were used to perform a meta-analysis by gender. Reasons for studies exclusion are provided in Online Resource 6. Heterogeneity tests between the two time periods for both women and men showed no significant difference in the suicide rates between the periods (*p* = 0.94 and *p* = 0.90, respectively; see Table 3 and Online Resource 8). Five studies provided suicide data by age groups, but pooled estimates were not calculated because of variations in age classification criteria. There is no evidence of significant change between suicide rates for the pre- and during-pandemic periods according to age groups (see Online Resource 9).

**Table 3:** Estimated pre- and during-pandemic suicide rates/100,000 by gender

Gender	Pre-pandemic suicide rate (95% CI) (until February 2020)	During-pandemic suicide rate (95% CI) (from March 2020 to June 2021)
Women	7.51 (CI 4.76–10.27) Heterogeneity (*I*^2^ = 99.67%, *p* < 0.01)	7.36 (CI 4.05–10.67) Heterogeneity (*I*^2^ = 99.78%, *p* < 0.01)
Men	20.00 (CI 11.08–28.92) Heterogeneity (*I*^2^ = 99.93%, *p* < 0.01)	19.21 (CI 10.15–28.26) Heterogeneity (*I*^2^ = 99.94%, *p* < 0.01)

Only two studies [30, 31] provided suicide data by ethnicity, which precluded pooled estimations. A decrease in suicide among whites and an increase among non-whites (especially Asian) were noticed (see Online Resource 10). Only one study [32] provided suicide data by SDI, which showed a slight increase in suicide rate among people with high SDI and a plateau of suicide rate among people with middle and low SDI (see Online Resource 11).

### Publication bias and certainty of findings

Publication bias was detected by generating a funnel plot (see Online Resource 12). The certainty of the findings using the GRADE [28] framework is very low. Although there was no major imprecision and indirectness, the lack of information on respective data sources may have introduced biased estimates. Major inconsistency in estimates was probable due to a large variation in effect size, as evidenced by the large *I*^2^ value and Chi-square test (see Online Resource 13).

### Meta-regression

In our post hoc meta-regression analysis, we aimed to further explore the sources of heterogeneity among the included studies. Following the Cochrane Handbook guidelines [29], we limited the meta-regression to comparison groups comprising ten or more studies. Among the comparison groups considered, only the one involving WHO regions met this threshold, encompassing a total of 34 studies. The results of these meta-regression analyses, which can be found in Online Resource 14, revealed no statistically significant differences between the groups. This indicates that the variations in effect sizes observed across different WHO regions were not statistically significant when accounting for other covariates in the model. Furthermore, the joint test for all covariates, including the Knapp–Hartung modification, yielded a *p *value of 0.3227, indicating that the overall test for the model was not statistically significant. Therefore, the WHO region did not appear to be a significant source of heterogeneity in our study.

## Discussion

Overall, there was no evidence to suggest that suicide rates changed significantly during the pandemic, a finding which was consistent according to gender, age group, and geographical (WHO region). Our research provides unique contributions, despite the existence of a recently published study on the impact of the COVID-19 pandemic on suicide rates. Our systematic review includes a larger number of papers, offering a more comprehensive analysis of the literature. In addition, we encompassed data from a wider range of countries/locations, providing a diverse perspective. Furthermore, our rigorous evaluation of suicide data sources enhances the reliability and validity of our findings.

This finding is in accordance with other published studies [22, 24, 33]. Pirkis et al. included data from 21 countries, and a more recent study from the same author increased the number of countries analyzed to 33. The most recent study published on the topic [24] had 20 data samples collected until December 2022. All of them showed no evidence of significant change in suicide rates between the periods. Only one of them included analysis by subgroups [33], whose results are also in accordance with ours. Studies have looked into changes in suicide rates during infectious disease outbreaks. Considering previous epidemics and pandemics, a recent review [33] indicates that the 2003 SARS pandemic may have increased suicide cases in Hong Kong among vulnerable groups. Similar associations were noted during the Great Influenza Epidemic and Russian influenza [34, 35].

Despite initial concerns about the potential negative effects of the pandemic on suicide risk factors such as mental health issues, domestic violence, financial stressors, and access to lethal means, only a few studies [36–38] have shown evidence of increased suicide incidence rates during the pandemic. Possible explanations for the lack of a rise in suicide rates include the economic support provided by the governments to mitigate the negative effects of lockdown and temporary closure of non-essential businesses [22, 39–44], which increased the unemployment rate (an important risk factor of suicide). Another relevant factor, noticed especially in high-income countries, is the boost in mental health care [22, 30, 39, 40, 44–46]. Either patients with previously diagnosed mental health conditions and people experiencing depression, loneliness and suicidal thoughts for the first time, both groups were well looked after with the maintenance of mental health facilities or the creation of telehealth services and special suicide helplines. A third factor that several studies say it may have contributed to prevent increased suicide rates is the strengthening of family, friendship, and community bonds. The feeling of unity in the face of a “bigger challenge” and the behavior of empathy, companionship, and solidarity, also known as social cohesion, especially with people at risk, were repeatedly cited [22, 30, 44, 47–51]. The fourth most cited reason was decreased stress and pressure related to work life balance due to remote working [22, 41, 44, 48].

Despite finding no change in rates of suicide during the pandemic, it is important to note that some populations may still be at increased risk, such as those with pre-existing mental health conditions or those experiencing significant financial or social stressors. With governments withdrawing economic support and a possible recession, lag effect awareness is crucial. Evidence suggests a rise in suicide cases during times of economic downturn and following natural disasters [52, 53]. New studies and improvements in suicide prevention are crucial. Continued monitoring and support for those at risk is essential.

We found no significant differences according to gender or age group. However, women may have been more vulnerable to lockdowns and restrictive measures due to their presence in highly affected sectors, such as service industries, and irregular and precarious jobs [29, 32, 36]. Women who had to work from home while managing children, particularly in areas with high gender inequality or domestic violence, faced additional challenges [54–56]. In contrast, men have been particularly affected by economic consequences of the pandemic and may be less likely to seek help for mental health issues [57, 58].

We were unable to detect if the COVID-19 pandemic affected age groups differently. Rises and falls were observed in almost all age groups in different areas. Children in China might be affected due to academic stress, domestic violence, and social isolation [59, 60]. Elderly people, who generally have higher suicide rates, may have faced increased family conflicts [60, 61].

### Strengths and limitations

High quality and widely available suicide data are rare around the globe. Around two-thirds of the nations do not provide sufficient or reliable information on suicide mortality [62]. One of the strengths of this study is a comprehensive systematic review providing pooled estimates to inform temporal trends in suicide rates during the COVID-19 pandemic in over 45 areas, including whole countries, cities, counties, and regions, covering a longer duration. However, the study has several limitations. Publication bias is one such limitation as we only included English language, full-text articles, potentially overlooking relevant non-English language studies and data from grey literature and organizational websites. Not all WHO geographic regions were analyzed. The inconsistency of suicide data sources across studies is inherently a methodological limitation. The lack of clarity on defining a population base and standardized definition of suicide added to the methodological challenge. We attempted to address some of these methodological challenges through non-inclusion of discrepant data in the analysis and stating the risk of bias in our study.

## Conclusion

The study showed no significant increase in suicide incidence rate during the COVID-19 pandemic period compared to the pre-pandemic period following a comprehensive scrutiny of individual studies relating to suicide data sources. One such source is real-time surveillance data. However, given the limited time frame of the included studies, longer term studies are necessary to fully understand the total effect of the pandemic on suicide rates. It is important to note that the absence of a pandemic-related increase in suicides does not justify relaxing suicide prevention policies or halting improvements in suicide data reporting.

## Supplementary Information

Below is the link to the electronic supplementary material.

Supplementary file1 (DOCX 299 KB)

## References

[1] Who director-general's opening remarks at the media briefing on COVID-19 - 11 march 2020. World Health Organization. https://www.who.int/director-general/speeches/detail/who-director-general-s-opening-remarks-at-the-media-briefing-on-covid-19---11-march-2020. Published March 11, 2020. Accessed April 10, 2023.

[2] Who coronavirus (COVID-19) dashboard. World Health Organization. https://covid19.who.int/. Accessed April 10, 2023.

[3] Ilo Monitor on the world of work. 9th edition. Monitor on the world of work. 9th edition. https://www.ilo.org/global/publications/books/WCMS_845642/lang--en/index.htm. Published May 23, 2022. Accessed April 10, 2023.

[4] OECD Economic Outlook (2021) interim report September 2021. OECD Economic Outlook. 10.1787/490d4832-en

[5] Nepogodiev D, Abbott TE, Ademuyiwa AO et al (2022) Projecting COVID-19 disruption to elective surgery. The Lancet 399(10321):233–234. 10.1016/s0140-6736(21)02836-1 10.1016/S0140-6736(21)02836-1 PMC8676422 34922640

[6] Elective surgery cancellations due to the COVID-19 pandemic (2020) global predictive modelling to inform surgical recovery plans. Br J Surg. 10.1002/bjs.11746 10.1002/bjs.11746 PMC7272903 32395848

[7] Kendzerska T, Zhu DT, Gershon AS et al (2021) The Effects of the Health System Response to the COVID-19 Pandemic on Chronic Disease Management: A Narrative Review. RMHP 14:575–584. 10.2147/rmhp.s293471 10.2147/RMHP.S293471 PMC7894869 33623448

[8] Singh K, Xin Y, Xiao Y et al (2022) Impact of the COVID-19 Pandemic on Chronic Disease Care in India, China, Hong Kong, Korea, and Vietnam. Asia Pac J Public Health 34(4):392–400. 10.1177/10105395211073052 35067078 10.1177/10105395211073052 PMC9133173

[9] UNICEF. Education Disrupted: the second year of the COVID-19 pandemic and school closures. September 2021. [cited may 30 2022] Available from: https://data.unicef.org/wp-content/uploads/2021/09/Education-disrupted-School-closures-brochure-2021.pdf

[10] Aknin LB, Andretti B, Goldszmidt R et al (2022) Policy stringency and mental health during the COVID-19 pandemic: a longitudinal analysis of data from 15 countries. The Lancet Public Health 7(5):e417–e426. 10.1016/s2468-2667(22)00060-3 35461592 10.1016/S2468-2667(22)00060-3 PMC9023007

[11] Xiong J, Lipsitz O, Nasri F et al (2020) Impact of COVID-19 pandemic on mental health in the general population: A systematic review. J Affect Disord 277:55–64. 10.1016/j.jad.2020.08.001 32799105 10.1016/j.jad.2020.08.001 PMC7413844

[12] Vindegaard N, Benros ME (2020) COVID-19 pandemic and mental health consequences: Systematic review of the current evidence. Brain Behav Immun 89:531–542. 10.1016/j.bbi.2020.05.048 32485289 10.1016/j.bbi.2020.05.048 PMC7260522

[13] Wu T, Jia X, Shi H et al (2021) Prevalence of mental health problems during the COVID-19 pandemic: A systematic review and meta-analysis. J Affect Disord 281:91–98. 10.1016/j.jad.2020.11.117 33310451 10.1016/j.jad.2020.11.117 PMC7710473

[14] Eyles E, Moran P, Okolie C et al (2021) Systematic review of the impact of the COVID-19 pandemic on suicidal behaviour amongst health and social care workers across the world. J Affect Disord Rep. 10.1016/j.jadr.2021.100271 34841385 10.1016/j.jadr.2021.100271 PMC8607051

[15] Turecki G, Brent DA, Gunnell D et al (2019) Suicide and suicide risk. Nat Rev Dis Primers. 10.1038/s41572-019-0121-0 31649257 10.1038/s41572-019-0121-0

[16] Oyesanya M, Lopez-Morinigo J, Dutta R (2015) Systematic review of suicide in economic recession. WJP 5(2):243. 10.5498/wjp.v5.i2.243 26110126 10.5498/wjp.v5.i2.243 PMC4473496

[17] Kõlves K, Kõlves KE, De Leo D (2013) Natural disasters and suicidal behaviours: A systematic literature review. J Affect Disord 146(1):1–14. 10.1016/j.jad.2012.07.037 22917940 10.1016/j.jad.2012.07.037

[18] Jankovic J, Bremner S, Bogic M et al (2013) Trauma and suicidality in war affected communities. Eur psychiatr 28(8):514–520. 10.1016/j.eurpsy.2012.06.001 10.1016/j.eurpsy.2012.06.001 22986125

[19] Bosnar A, Stemberga V, Coklo M et al (2005) Suicide and the war in Croatia. Forensic Sci Int 147:S13–S16. 10.1016/j.forsciint.2004.09.086 15694719 10.1016/j.forsciint.2004.09.086

[20] Music E, Jacobsson L, Salander RE (2014) Suicide in Bosnia and Herzegovina and the City of Sarajevo. Crisis 35(1):42–50. 10.1027/0227-5910/a000232 24197489 10.1027/0227-5910/a000232

[21] Zortea TC, Brenna CTA, Joyce M et al (2021) The Impact of Infectious Disease-Related Public Health Emergencies on Suicide, Suicidal Behavior, and Suicidal Thoughts. Crisis 42(6):474–487. 10.1027/0227-5910/a000753 33063542 10.1027/0227-5910/a000753 PMC8689932

[22] Pirkis J, John A, Shin S et al (2021) Suicide trends in the early months of the COVID-19 pandemic: an interrupted time-series analysis of preliminary data from 21 countries. The Lancet Psychiatry 8(7):579–588. 10.1016/s2215-0366(21)00091-2 33862016 10.1016/S2215-0366(21)00091-2 PMC9188435

[23] Webb RT, John A, Knipe D et al (2022) Has the COVID-19 pandemic influenced suicide rates differentially according to socioeconomic indices and ethnicity? More evidence is needed globally. Epidemiol Psychiatr Sci. 10.1017/s2045796022000543 36217667 10.1017/S2045796022000543 PMC9579839

[24] Yan Y, Hou J, Li Q, Yu NX (2023) Suicide before and during the COVID-19 Pandemic: A Systematic Review with Meta-Analysis. IJERPH 20(4):3346. 10.3390/ijerph20043346 36834037 10.3390/ijerph20043346 PMC9960664

[25] Moher D, Liberati A, Tetzlaff J, Altman DG (2010) Preferred reporting items for systematic reviews and meta-analyses: The PRISMA statement. Int J Surg 8(5):336–341. 10.1016/j.ijsu.2010.02.007 20171303 10.1016/j.ijsu.2010.02.007

[26] Munn Z, Moola S, Lisy K, Riitano D, Tufanaru C (2015) Methodological guidance for systematic reviews of observational epidemiological studies reporting prevalence and cumulative incidence data. Int J Evid Based Healthc 13(3):147–153. 10.1097/xeb.0000000000000054 26317388 10.1097/XEB.0000000000000054

[27] Nyaga VN, Arbyn M, Aerts M (2014) Metaprop: a Stata command to perform meta-analysis of binomial data. Arch Public Health. 10.1186/2049-3258-72-39 25810908 10.1186/2049-3258-72-39 PMC4373114

[28] Schünemann H, Brożek J, Guyatt G, Oxman A, editors. GRADE handbook for grading quality of evidence and strength of recommendations. Updated October 2013. The GRADE Working Group, 2013. Available from guidelinedevelopment.org/handbook

[29] Deeks JJ, Higgins JPT, Altman DG (editors). Chapter 10: Analysing data and undertaking meta-analyses. In: Higgins JPT, Thomas J, Chandler J, Cumpston M, Li T, Page MJ, Welch VA (editors). Cochrane Handbook for Systematic Reviews of Interventions, version 6.3 (updated February 2022). Cochrane, 2022. Available at www.training.cochrane.org/handbook.

[30] Bray MJC, Daneshvari NO, Radhakrishnan I et al (2021) Racial Differences in Statewide Suicide Mortality Trends in Maryland During the Coronavirus Disease 2019 (COVID-19) Pandemic. JAMA Psychiat 78(4):444. 10.1001/jamapsychiatry.2020.3938 10.1001/jamapsychiatry.2020.3938 PMC7745133 33325985

[31] Mitchell TO, Li L (2021) State-Level Data on Suicide Mortality During COVID-19 Quarantine: Early Evidence of a Disproportionate Impact on Racial Minorities. Psychiatry Res. 10.1016/j.psychres.2020.113629 33290944 10.1016/j.psychres.2020.113629

[32] Arya V, Page A, Spittal MJ et al (2022) Suicide in India during the first year of the COVID-19 pandemic. J Affect Disord 307:215–220. 10.1016/j.jad.2022.03.066 35395323 10.1016/j.jad.2022.03.066 PMC8983610

[33] Pirkis J, Gunnell D, Shin S et al (2022) Suicide numbers during the first 9–15 months of the COVID-19 pandemic compared with pre-existing trends: An interrupted time series analysis in 33 countries. Clin Med. 10.1016/j.eclinm.2022.101573 10.1016/j.eclinm.2022.101573 PMC9344880 35935344

[34] Wasserman IM (1992) The impact of epidemic, war, prohibition and media on suicide: United States, 1910–1920. Suicide Life Threat Behav 22(2):240–254 1626335

[35] Honigsbaum M (2010) The Great Dread: Cultural and Psychological Impacts and Responses to the “Russian” Influenza in the United Kingdom, 1889–1893. Social History of Medicine 23(2):299–319. 10.1093/shm/hkq011

[36] Acharya B, Subedi K, Acharya P, Ghimire S. Association between COVID-19 pandemic and the suicide rates in Nepal. Leong C, ed. PLoS ONE. 2022;17(1):e0262958. doi:10.1371/journal.pone.0262958 10.1371/journal.pone.0262958 PMC8786170 35073377

[37] Rogalska A, Syrkiewicz-Świtała M (2022) COVID-19 and Mortality, Depression, and Suicide in the Polish Population. Front Public Health. 10.3389/fpubh.2022.854028 35372182 10.3389/fpubh.2022.854028 PMC8965814

[38] Borges G, Garcia JA, Pirkis J et al (2022) A state level analyses of suicide and the COVID-19 pandemic in Mexico. BMC Psychiatry. 10.1186/s12888-022-04095-8 35810285 10.1186/s12888-022-04095-8 PMC9271255

[39] Leske S, Kõlves K, Crompton D, Arensman E, de Leo D (2021) Real-time suicide mortality data from police reports in Queensland, Australia, during the COVID-19 pandemic: an interrupted time-series analysis. Lancet Psychiatry 8(1):58–63. 10.1016/s2215-0366(20)30435-1 33212023 10.1016/S2215-0366(20)30435-1 PMC7836943

[40] Qin P, Mehlum L (2021) National observation of death by suicide in the first 3 months under COVID-19 pandemic. Acta Psychiatr Scand 143(1):92–93. 10.1111/acps.13246 33111325 10.1111/acps.13246

[41] Tanaka T, Okamoto S (2021) Increase in suicide following an initial decline during the COVID-19 pandemic in Japan. Nat Hum Behav 5(2):229–238. 10.1038/s41562-020-01042-z 33452498 10.1038/s41562-020-01042-z

[42] Knudsen AKS, Stene-Larsen K, Gustavson K et al (2021) Prevalence of mental disorders, suicidal ideation and suicides in the general population before and during the COVID-19 pandemic in Norway: A population-based repeated cross-sectional analysis. Lancet Reg Health Eur. 10.1016/j.lanepe.2021.100071 34557811 10.1016/j.lanepe.2021.100071 PMC8454837

[43] Barbic D, Scheuermeyer FX, Barbic SP, Honer WG (2021) Suicide Deaths in British Columbia during the First Wave of the COVID-19 Pandemic. Can J Psychiatry 66(9):830–831. 10.1177/07067437211018398 33998835 10.1177/07067437211018398 PMC8495302

[44] Efstathiou V, Stefanou MI, Siafakas N et al (2021) Suicidality and COVID-19: Suicidal ideation, suicidal behaviors and completed suicides amidst the COVID-19 pandemic (Review). Exp Ther Med. 10.3892/etm.2021.11030 34976149 10.3892/etm.2021.11030 PMC8674972

[45] McIntyre RS, Lui LM, Rosenblat JD et al (2021) Suicide reduction in Canada during the COVID-19 pandemic: lessons informing national prevention strategies for suicide reduction. J R Soc Med 114(10):473–479. 10.1177/01410768211043186 34551280 10.1177/01410768211043186 PMC8532219

[46] Sakamoto H, Ishikane M, Ghaznavi C, Ueda P (2021) Assessment of Suicide in Japan During the COVID-19 Pandemic vs Previous Years. JAMA Netw Open. 10.1001/jamanetworkopen.2020.37378 33528554 10.1001/jamanetworkopen.2020.37378 PMC7856546

[47] Appleby L, Richards N, Ibrahim S, Turnbull P, Rodway C, Kapur N (2021) Suicide in England in the COVID-19 pandemic: Early observational data from real time surveillance. Lancet Reg Health Eur. 10.1016/j.lanepe.2021.100110 34557817 10.1016/j.lanepe.2021.100110 PMC8454726

[48] Gerstner RM, Narváez F, Leske S et al (2022) Police-reported suicides during the first 16 months of the COVID-19 pandemic in Ecuador: A time-series analysis of trends and risk factors until June 2021. Lancet Reg Health Americas. 10.1016/j.lana.2022.100324 35912285 10.1016/j.lana.2022.100324 PMC9310552

[49] de la Torre-Luque A, Pemau A, Perez-Sola V, Ayuso-Mateos JL (2022) Suicide mortality in Spain in 2020: The impact of the COVID-19 pandemic. Rev Psiquiat Salud Mental. 10.1016/j.rpsm.2022.01.003 10.1016/j.rpsm.2022.01.003 PMC8809655 35132342

[50] Orellana JDY, de Souza MLP (2022) Excess suicides in Brazil: Inequalities according to age groups and regions during the COVID-19 pandemic. Int J Soc Psychiatry 68(5):997–1009. 10.1177/00207640221097826 35621004 10.1177/00207640221097826

[51] Ornell F, Benzano D, Borelli WV et al (2022) Differential impact in suicide mortality during the COVID-19 pandemic in Brazil. Br J Psych. 10.47626/1516-4446-2022-2581 10.47626/1516-4446-2022-2581 PMC9851756 35839315

[52] Chang SS, Stuckler D, Yip P, Gunnell D (2013) Impact of 2008 global economic crisis on suicide: time trend study in 54 countries. BMJ. 10.1136/bmj.f5239 24046155 10.1136/bmj.f5239 PMC3776046

[53] Chou YJ, Huang N, Lee CH et al (2003) Suicides after the 1999 Taiwan earthquake. Int J Epidemiol 32(6):1007–1014. 10.1093/ije/dyg296 14681266 10.1093/ije/dyg296

[54] Nivakoski S, Mascherini M (2021) Gender Differences in the Impact of the COVID-19 Pandemic on Employment, Unpaid Work and Well-Being in the EU. Inter Econ 56(5):254–260. 10.1007/s10272-021-0994-5 34629500 10.1007/s10272-021-0994-5 PMC8490839

[55] Chang Q, Yip PSF, Chen YY (2019) Gender inequality and suicide gender ratios in the world. J Affect Disord 243:297–304. 10.1016/j.jad.2018.09.032 30261445 10.1016/j.jad.2018.09.032

[56] Sánchez OR, Vale DB, Rodrigues L, Surita FG (2020) Violence against women during the COVID-19 pandemic: An integrative review. Int J Gynaecol Obstet 151(2):180–187. 10.1002/ijgo.13365 32880941 10.1002/ijgo.13365 PMC9087782

[57] Chen YY, Cai Z, Chang Q, Canetto SS, Yip PSF (2021) Caregiving as suicide-prevention: an ecological 20-country study of the association between men’s family carework, unemployment, and suicide. Soc Psychiatry Psychiatr Epidemiol 56(12):2185–2198. 10.1007/s00127-021-02095-9 33948679 10.1007/s00127-021-02095-9

[58] Pierce BS, Perrin PB, Tyler CM, McKee GB, Watson JD (2021) The COVID-19 telepsychology revolution: A national study of pandemic-based changes in US mental health care delivery. Am Psychol. 10.1037/amp0000722 32816503 10.1037/amp0000722

[59] Hoekstra PJ (2020) Suicidality in children and adolescents: lessons to be learned from the COVID-19 crisis. Eur Child Adolesc Psychiatry 29(6):737–738. 10.1007/s00787-020-01570-z 32488455 10.1007/s00787-020-01570-z PMC7266412

[60] Zheng XY, Tang SL, Ma SL et al (2021) Trends of injury mortality during the COVID-19 period in Guangdong, China: a population-based retrospective analysis. BMJ Open. 10.1136/bmjopen-2020-045317 34083336 10.1136/bmjopen-2020-045317 PMC8182756

[61] Coope C, Gunnell D, Hollingworth W et al (2014) Suicide and the 2008 economic recession: who is most at risk? Trends in suicide rates in England and Wales 2001–2011. Soc Sci Med 117:76–85. 10.1016/j.socscimed.2014.07.024 25054280 10.1016/j.socscimed.2014.07.024 PMC4151136

[62] Suicide worldwide in 2019. World Health Organization. https://www.who.int/publications-detail-redirect/9789240026643. Published 2021. Accessed April 10, 2023

