# Correction: Suicide rates before and during the COVID-19 pandemic: a systematic review and meta-analysis

**DOI:** 10.1007/s00127-024-02651-z

**Published:** 2024-03-13

**Authors:** Ana Paula da Cunha Varella, Eve Griffin, Ali Khashan, Zubair Kabir

**Affiliations:** 1 School of Public Health-UCC, University College Cork, Western Gateway Building (4th Floor), Cork City, co., Cork, Ireland; 2 National Suicide Research Foundation, Cork, Ireland

**Correction: Social Psychiatry and Psychiatric Epidemiology**

10.1007/s00127-024-02617-1

In this article the author name “Ali Khashan” was incorrectly written as “Ali Kashan”.

The original article has been corrected.

