# Supplementary material for: Suicide rates before and during the COVID-19 pandemic: a systematic review and meta-analysis
Source: Soc Psychiatry Psychiatr Epidemiol. 2024 Feb 14;59(11):1897–905. doi: 10.1007/s00127-024-02617-1 (PMC11522181; doi:10.1007/s00127-024-02617-1)
Supplement: Supplementary file 1 — Supplementary file1 (DOCX 299 KB) [file 127_2024_2617_MOESM1_ESM.docx]

**SUPPLEMENT FILE**

**Title:** Suicide rates before and during the COVID-19 pandemic: a systematic review and meta-analysis

**Journal:** Social Psychiatry and Psychiatric Epidemiology

**Authors:**

Ana Paula da Cunha Varella, MPH

School of Public Health, University College Cork, Cork, Ireland

Society of Epidemiologic Research, Eagle, Idaho, USA

anavarellaa@gmail.com

Eve Griffin, PhD

School of Public Health, University College Cork, Cork, Ireland

National Suicide Research Foundation, Cork, Ireland

Zubair Kabir, PhD

School of Public Health, University College Cork, Cork, Ireland

[Online Resource 1: Detailed Search Strategy 2](#_35nkun2)

[Online Resource 2: Excluded studies with reasons (second step of study selection) 4](#_1v1yuxt)

[Online Resource 3: Quality Appraisal 6](#_4f1mdlm)

[Online Resource 4: Data transformation 8](#_19c6y18)

[Online Resource 5: Characteristics of the included studies 10](#_1664s55)

[Online Resource 6: Excluded studies from meta-analysis 15](#_25b2l0r)

[Online Resource 7: Forest plots of the pooled suicide rate for the pre pandemic period (A) and the during pandemic period (B) by WHO region 17](#_kgcv8k)

[Online Resource 8: Forest plot of the pooled suicide rate for the pre and during pandemic periods among women (A) and men (B) 19](#_34g0dwd)

[Online Resource 9: Estimated suicide rates (95% CI)/100,000 by age groups* 21](#_2koq656)

[Online Resource 10: Estimated suicide rates (95% CI)/100,000 by ethnicity 23](#_zu0gcz)

[Online Resource 11: Estimated Suicide rates (95% CI)/100,000 by socio-demographic index (SDI) 24](#_3jtnz0s)

[Online Resource 12: Funnel plot of studies investigating suicide rates in the pre and during Covid-19 pandemic periods 25](#_1yyy98l)

[Online Resource 13: GRADE Evidence Profile of all Observational Studies included in the Systematic Review 26](#_1d96cc0)

[Online Resource 14: Results of the meta-regression between WHO regions 27](#_z8tugueechtm)

[References 28](#_338fx5o)

#

#

#

#

#

#

#

#

#

#

#

#

#

#

# Online Resource 1: Detailed Search Strategy

The specific search terms and results for each database (conducted on June 1st, 2022) are shown below:

PubMed

Search:

#1: "suicide"[MeSH Terms] OR "suicid*"[Title/Abstract] OR "suicide rate*"[Title/Abstract] OR "suicide trend*"[Title/Abstract]

#2: "covid 19"[MeSH Terms] OR "sars cov 2"[MeSH Terms]

#3: "risk*"[Title] OR "depressi*"[Title] OR "anxiety"[Title] OR "alcohol"[Title] OR "burnout"[Title] OR "drug*"[Title] OR "review"[Title]

#4 (#1 AND #2 NOT #3): (((((suicide[MeSH Terms]) OR (suicid*[Title/Abstract])) OR ("suicide rate*"[Title/Abstract])) OR ("suicide trend*"[Title/Abstract])) AND ((COVID-19[MeSH Terms]) OR (SARS-CoV-2[MeSH Terms]))) NOT (((((((risk*[Title]) OR (depressi*[Title])) OR (anxiety[Title])) OR (alcohol[Title])) OR (burnout[Title])) OR (drug*[Title])) OR (review[Title]))

Results: 695

Web of Science

Topic search includes title, abstract, author keywords and Keyword Plus.

Search:

#1 Covid-19 OR Sars-Covd-2 (Topic)

#2 suicid* (Topic)

#3 trend* OR rate* OR prevalence OR incidence (Topic)

#4 review* (Topic)

#5 (#1 AND #2 AND #3 NOT #4): (((TS=(Covid-19 OR Sars-Cov-2)) AND TS=(suicid*)) AND TS=(trend* OR rate* OR prevalence OR incidence)) NOT TS=(review*)

Results: 561

Scopus

Search:

#1 covid AND suicid* (Title/Abstract/Keyword)

#2 rate* OR trend* OR prevalence OR incidence (Title/Abstract/Keyword)

#3 ideation (Title/Abstract/Keyword)

#4 (#1 AND #2 AND NOT #3): ( TITLE-ABS-KEY ( covid AND suicid* ) AND TITLE-ABS-KEY ( rate* OR trend* OR prevalence OR incidence ) AND NOT TITLE-ABS-KEY ( ideation ) )

Results: 347

PsychINFO

Search:

#1 suicid* (Full text)

#2 covid OR sars cov 2 (Full text)

#3 trend* OR rate* OR prevalence OR incidence (Abstract)

#4 (#1 AND #2 AND #3) TX suicid* AND TX ( covid OR sars cov 2 ) AND AB ( trend* OR rate* OR prevalence OR incidence )

Results: 210

Online resource 1: the search strategy used for each database (PubMed, Web of Science, Scopus and PsychINFO) are detailed. It contains the terms used in the search, the steps of the search and how many results were found.

#

#

#

#

#

#

#

#

#

#

#

#

#

#

#

#

#

#

#

#

#

#

#

#

#

#

# Online Resource 2: Excluded studies with reasons (second step of study selection)

The second step of the study selection process is the full text examination of the eligibility criteria. Two hundred and one studies were evaluated in this phase and 167 were excluded. The reasons for exclusion are explained in the table below.

| **Reason for exclusion** | **Explanation** | **Number of studies** | **Studies** |
| --- | --- | --- | --- |
| Inadequate comparison | No comparison group Comparisons using projected rather than actual incidences | 8 | Lin, Isnar, Daria, Taira, Koda, Kaggwa, Osaki, Orui |
| Inadequate data sources | Unspecified  Newspaper reports | 6 | Singh, Fuse-Nagase, Faust, Garbarino, Bellman, Karakasi |
| Insufficient data | Studies which do not provide the prevalence of suicide, neither sufficient data to calculate it | 13 | Page, Dwyer, Isumi, Sakelliadis, Calderon-Anyosa (a), Faust, Ueda, Eguchi, Rossom, Su, Radeloff, Grande, Calderon Anyosa (b) |
| Other outcomes | Suicide ideation  Other self-harm behaviors including suicide attempts | 9 | Kim, Kang, Kim, Valdez-Santiago, Jollant, Ferrando, Gétaz, Prados-Ojeda, Steeg |
| Out of scope | If the study fits into 2 or more of the previous categories | 116 | Standish, Brenner, Dsouza, Choudhury, Sign, Travis-Lumer, Kingston, Pompili, Devitt, Kallakuri, Ehlman, Buschmann, Thakur, Charlier, dos Santos, The Lancet Psychiatry, Wand, Tandon, Panigrani, Monjur, Menon, Jollant, Amerio, Mamun, Seposo, Wise, Wand, Jefsen, Joseph, Ontiveros, Sher, Kapur, Hewson, Rana, Koda, Rogers, Philip, Anzai, Shoib, Houry, Swain, Nooraeen, Ward, Marutani, Fitzpatrick, Carlin, Kar, Page, Shrestha, Karakasi, García-Ullá, Carison, Sher, Schaughency, Fusar-Poli, Chung, Singh, Mi, Galea, Goyal, Mei, Guraj, Poorolajal, Leipinik, Sengupta, Duarte, Saeed, Laboe, Nichter, Hou, Ramchand, McIntyre, Samson, Sher, Jurblum, Gonzalez, Guille, Menon, Inoue, Saavedra-López, Hawton, Demuthova, Park, Inoue, Balachandran, Batyrgareieva, Banda, Amadéo, Awan, Courtet, Zalsman, Garnett, Uvais, Bellizzi, Klomek, Sher, Kapilam, Ball, Bastiampillai, Challinor, Chidiebere, Zortea, Sher, Fushimi, Khan, Vijayalumar, Mamun, Hernández-Calle, John, Nomura, Hughes, Brenner, Dready, Legler, Appleby, Moser |
| Other languages other than English | Spanish, Russian, Hungarian, Dutch and German | 7 | Osváth. González-Rodrígues, de Winter, Kekelidze, Ozsoz, Cardenas, Kekelidze |
| Full text unavailable | Free access unavailable | 8 | Pokhrel, Abi Zeid Daou, Almaghrebi, Adler, Sahu, Patel, Partonen, Koilara |

Online resource 2: the table shows the reason for exclusion of the study, followed by its explanation, the number of studies excluded for that reason and the first author's last name. The reasons are: inadequate comparison (no comparison group or comparisons using projected rather than actual incidences, 8 studies), inadequate data source (unspecified or newspaper reports, 6 studies), insufficient data (studies which do not provide the prevalence of suicide, neither sufficient data to calculate it, 13 studies), other outcomes (Suicide ideation or other self-harm behaviors including suicide attempts, 9 studies), out of scope (if the study fits into 2 or more of the previous categories, 116 studies), other languages (7 studies) and full text unavailable (8 studies).

#

#

#

#

#

#

# Online Resource 3: Quality Appraisal

The table below shows the results of the quality appraisal of each included study using the The Joanna Briggs Institute Critical Appraisal Tool For Studies Reporting Prevalence Data (JBI).

| **Study number** | **Reviewer** | **Date** | **Author** | **Year** | **Q1** | **Q2** | **Q3** | **Q4** | **Q5** | **Q6** | **Q7** | **Q8** | **Q9** | **Overall appraisal** |
| --- | --- | --- | --- | --- | --- | --- | --- | --- | --- | --- | --- | --- | --- | --- |
| **1** | AV | 22/06/2022 | Sakamoto, H^1^ | 2021 | Yes | N/A | Yes | Yes | N/A | Yes | Yes | Yes | N/A | Include |
| **2** | AV | 22/06/2022 | Acharya, B^2^ | 2022 | Yes | N/A | Yes | Yes | N/A | Yes | Yes | Yes | N/A | Include |
| **3** | AV | 22/06/2022 | Odd, D^3^ | 2021 | Yes | N/A | Yes | Yes | N/A | Yes | Yes | Yes | N/A | Include |
| **4** | AV | 22/06/2022 | Deisenhammer, D^4^ | 2021 | Yes | N/A | Yes | Yes | N/A | Unclear | Unclear | Yes | N/A | Include |
| **5** | AV | 22/06/2022 | Matsumoto, R^5^ | 2021 | Yes | N/A | Yes | Yes | N/A | Unclear | Unclear | Yes | N/A | Include |
| **6** | AV | 22/06/2022 | Inoue, K^6^ | 2022 | Yes | N/A | Yes | Yes | N/A | Unclear | Unclear | Yes | N/A | Include |
| **7** | AV | 22/06/2022 | Buciuta, M^7^ | 2022 | Yes | N/A | Yes | Yes | N/A | Yes | Yes | Yes | N/A | Include |
| **8** | AV | 22/06/2022 | Watanabe, M^8^ | 2022 | Yes | N/A | Yes | Yes | N/A | Unclear | Unclear | Yes | N/A | Include |
| **9** | AV | 22/06/2022 | Tanaka, T^9^ | 2021 | Yes | N/A | Yes | Yes | N/A | Unclear | Unclear | Yes | N/A | Include |
| **10** | AV | 22/06/2022 | Fushimi, M^10^ | 2022 | Yes | N/A | Yes | Yes | N/A | Unclear | Unclear | Yes | N/A | Include |
| **11** | AV | 22/06/2022 | Sánchez, R^11^ | 2021 | Yes | N/A | Yes | Yes | N/A | Unclear | Unclear | Yes | N/A | Include |
| **12** | AV | 22/06/2022 | Qin, P^12^ | 2021 | Yes | N/A | Yes | Yes | N/A | Unclear | Unclear | Yes | N/A | Include |
| **13** | AV | 22/06/2022 | Clapperton, A^13^ | 2021 | Yes | N/A | Yes | Yes | N/A | Yes | Yes | Yes | N/A | Include |
| **14** | AV | 22/06/2022 | Calati, R^14^ | 2021 | Yes | N/A | 30/ | Yes | N/A | Yes | Yes | Yes | N/A | Include |
| **15** | AV | 22/06/2022 | Knudsen, A^15^ | 2021 | Yes | N/A | Yes | Yes | N/A | Yes | Yes | Yes | N/A | Include |
| **16** | AV | 22/06/2022 | Bray, M^16^ | 2021 | Yes | N/A | Yes | Yes | N/A | Yes | Yes | Yes | N/A | Include |
| **17** | AV | 22/06/2022 | Leske, S^17^ | 2021 | Yes | N/A | Yes | Yes | N/A | Yes | Yes | Yes | N/A | Include |
| **18** | AV | 22/06/2022 | Mitchell, T^18^ | 2021 | Yes | N/A | Yes | Yes | N/A | Yes | Yes | Yes | N/A | Include |
| **19** | AV | 22/06/2022 | Schleihauf, E^19^ | 2022 | Yes | N/A | Yes | Yes | N/A | Yes | Yes | Yes | N/A | Include |
| **20** | AV | 22/06/2022 | Barbic, D^20^ | 2021 | Yes | N/A | Yes | Yes | N/A | Yes | Yes | Yes | N/A | Include |
| **21** | AV | 22/06/2022 | Appleby, L^21^ | 2021 | Yes | N/A | Yes | Yes | N/A | Yes | Yes | Yes | N/A | Include |
| **22** | AV | 22/06/2022 | Arya, V^22^ | 2022 | Yes | N/A | Yes | Yes | N/A | Unclear | Unclear | Yes | N/A | Include |
| **23** | AV | 24/06/2022 | McIntyre, R^23^ | 2021 | Yes | N/A | Yes | Yes | N/A | Yes | Yes | Yes | N/A | Include |
| **24** | AV | 24/06/2022 | Stene-Larsen, K^24^ | 2022 | Yes | N/A | Yes | Yes | N/A | Unclear | Unclear | Yes | N/A | Include |
| **25** | AV | 24/06/2022 | Pirkis, J^25^ | 2021 | Yes | N/A | Yes | Yes | N/A | Yes | Yes | Yes | N/A | Include |
| **26** | AV | 24/06/2022 | Chen, Y^26^ | 2022 | Yes | N/A | Yes | Yes | N/A | Yes | Yes | Yes | N/A | Include |
| **27** | AV | 24/06/2022 | Pérez, V^27^ | 2022 | Yes | N/A | Yes | Yes | N/A | Unclear | Unclear | Yes | N/A | Include |
| **28** | AV | 24/06/2022 | Wollschläger, D^28^ | 2021 | Yes | N/A | Yes | Yes | N/A | Yes | Yes | Yes | N/A | Include |
| **29** | AV | 24/06/2022 | Ando, M^29^ | 2022 | Yes | N/A | Yes | Yes | N/A | Unclear | Unclear | Yes | N/A | Include |
| **30** | AV | 24/06/2022 | Yorsaeng, R^30^ | 2022 | Yes | N/A | Yes | Yes | N/A | Unclear | Unclear | Yes | N/A | Include |
| **31** | AV | 24/06/2022 | Kim, A^31^ | 2021 | Yes | N/A | Yes | Yes | N/A | Unclear | Unclear | Yes | N/A | Include |
| **32** | AV | 24/06/2022 | Behera, C^32^ | 2021 | Yes | N/A | Yes | Yes | N/A | Yes | Yes | Yes | N/A | Include |
| **33** | AV | 24/06/2022 | Nomura, S^33^ | 2021 | Yes | N/A | Yes | Yes | N/A | Unclear | Unclear | Yes | N/A | Include |
| **34** | AV | 24/06/2022 | Zheng, X^34^ | 2021 | Yes | N/A | Yes | Yes | N/A | Yes | Yes | Yes | N/A | Include |

#

Online resource 3: the table contains the initials of the author who performed the quality appraisal, the date, the study’s author’s last name, the year of publication and the answer to each question. Questions 2, 5 and 9 were considered not applicable. Answers of questions 1, 3, 4 and 8 were “yes” to all studies. Answers of questions 6 and 7 were marked “unclear” for a few studies.

# Online Resource 4: Data transformation

Data reported in frequencies other than annual was transformed to annual values. In order to do that, the 12 month-period mean number of suicide cases was calculated. Examples of how the annual data were calculated for the 3 other categories are shown below:

*Category 2: monthly data - Acharya*^2^ *(Nepal)*

Pre pandemic period: Jul 2017 - Mar 2020 (33 months)

During pandemic period: Apr 2020 - Jun 2021 (15 months)

Data provided by the study: monthly number of suicide cases (as shown by Table 1).

Table 1: Number of suicide cases by month in Nepal (2017-2021)

| **Month** | **2017** | **2018** | **2019** | **2020** | **2021** |
| --- | --- | --- | --- | --- | --- |
| **Jan** | - | 368 | 413 | 394 | 512 |
| **Feb** | - | 409 | 439 | 425 | 512 |
| **Mar** | - | 446 | 470 | 459 | 543 |
| **Apr** | - | 482 | 533 | 541 | 589 |
| **May** | - | 494 | 598 | 624 | 629 |
| **Jun** | - | 569 | 604 | 818 | 732 |
| **Jul** | 432 | 548 | 533 | 838 | - |
| **Aug** | 413 | 436 | 497 | 661 | - |
| **Sep** | 422 | 422 | 478 | 588 | - |
| **Oct** | 435 | 437 | 446 | 550 | - |
| **Nov** | 397 | 457 | 468 | 544 | - |
| **Dec** | 359 | 442 | 418 | 526 | - |

Pre pandemic total number of cases: 15,143

Pre pandemic 12-month period mean number of cases: (15,143/33)*12 = 5,506.54

During pandemic total number of cases: 9,207

During pandemic 12-month period mean number of cases: (9,207/15)*12 = 7,365.60

*Category 3: same months per year - Deisenhammer*^4^ *(Tyrol, Austria)*

Pre pandemic period: April 1st - Sept 30th 2018 and 2019 (12 months)

During pandemic period: April 1st - Sept 30th 2020 (6 months)

Data provided by the study: number of suicide cases per period per year (as shown by table 2)

Table 2: Number of suicide cases in Tyrol, Austria

|  | 2018 (April 1st - Sept 30th) | 2019 (April 1st - Sept 30th) | 2020 (April 1st - Sept 30th) |
| --- | --- | --- | --- |
| Cases | 66 | 62 | 45 |

Pre pandemic 12-month period mean number of cases: 66 (6 months in 2018) + 62 (6 months in 2019) = 128

During pandemic 12-month period mean number of cases: 45*2 = 90

*Category 4: other frequency - Clapperton*^13^ *(Queensland, Victoria and Tasmania, Australia)*

Pre pandemic period: January 2017 to January 2020 (37 months)

During pandemic period: February 2020 to August 2020 (7 months)

Data provided by the study: total number os suicide cases per period (as shown by table 3)

Table 3: Number of suicide cases in Queensland, Victoria and Tasmania, Australia

|  | January 2017 to January 2020 | February 2020 to August 2020 |
| --- | --- | --- |
| Cases | 4,878 | 913 |

Pre pandemic 12-month period mean number of cases: (4,878/37)*12 = 1,582.05

During pandemic 12-month period mean number of cases: (913/7)*12 = 1,565.14

#

#

#

#

#

#

#

#

#

#

#

#

#

#

#

#

#

#

#

#

# Online Resource 5: Characteristics of the included studies

| **Author** | **Year Publication** | **Place** | **Population** | **Definition of suicide** | **Pre pandemic period** | **During pandemic period** | **Frequency of data report** | **Data source - suicide** | **Data source population data** | **Report data by gender** | **Report data by age groups** |
| --- | --- | --- | --- | --- | --- | --- | --- | --- | --- | --- | --- |
| Sakamoto, H^1^ | 2021 | Japan | All people | Not specified | Jan - Nov 2016-2019 | Jan - Nov 2020 | Monthly | Japanese Ministry of Health Labor and Welfare | Population Census of Japan | Yes | Yes |
| Acharya, B^2^ | 2022 | Nepal | All people | Not specified | July 17 - Mar 20 | Apr 20 - Jun 21 | Monthly | Nepal Police Headquarter | Not specified | No | No |
| Odd, D^3^ | 2021 | England | <18yo | Not specified | Apr - Dec 19 | Apr - Dec 20 | Those 9 months per year | National Child Mortality Database | Office for National Statistics | Yes | No |
| Deisenhammer, E^4^ | 2021 | Tyrol, Austria | All people | Not specified | April 1st - Sept 30th 2006-2019 | April 1st - Sept 30th 2020 | Those 6 months per year | Tyrol Suicide Register | Not specified | No | No |
| Matsumoto, R^5^ | 2021 | Japan | All people | Not specified | 2017-2019 | 2020-2021 (until Jun) | Monthly | Japanese Ministry of Health Labor and Welfare | Regional Statistics Database (RSD) | Yes | No |
| Inoue, K^6^ | 2022 | Japan | All people | Not specified | 2019 | 2020 | Annually | Japanese Ministry of Health Labor and Welfare | Population data was not provided | Yes | Yes |
| Buciuta, M^7^ | 2022 | Romania | All people | ICD X7x | 2016-2019 | 2020 | Annual | Institute of Forensic Medicine Cluj | Population data was not provided | No | No |
| Tanaka, T^9^ | 2021 | Japan | All people | Not specified | Nov 2016 - Jan 2020 | First outbreak Feb-Jun2020  Second outbreak Jul-Oct 2020 | Per period | Japanese Ministry of Health Labor and Welfare | Not specified | No | No |
| Watanabe, M^8^ | 2022 | Japan | All people | Not specified | Not specified | Not specified | Annually | Japanese Ministry of Health Labor and Welfare | Population data was not provided | Yes | Yes |
| Fushimi, M^10^ | 2022 | Japan | All people | Not specified | Not specified | Not specified | Annually | Japanese Ministry of Health Labor and Welfare | Population data was not used | No | No |
| Sánchez, R^11^ | 2021 | Japan | All people | Not specified | 2018-2019 | Jan - Nov 2020 | Annually | Japanese Ministry of Health Labor and Welfare | Statistical Handbooks of Japan | No | Yes |
| Qin, P^12^ | 2021 | Norway | All people | Not specified | March - April 2017-2018 | March - April 2020 | Those 3 months per year and monthly | Norwegian Cause of Death Registry | Population data was not provided | Yes | Yes |
| Clapperton, A^13^ | 2021 | Australia | All people | Suspected suicides | Jan 2017 - Jan 2020 | Feb 2020 - Aug 2020 | Pre (37 months) x during (7 months) | interim Queensland Suicide Register (iQSR), Victorian Suicide Register (VSR) and Tasmanian Suicide Register (TSR) | Population data was not provided | Yes | Yes |
| Calati, R^14^ | 2021 | Milan | All people | Not specified | Jan 19 - Feb 20 | Mar 20 - Apr 21 | Monthly | Institute of Forensic Medicine | Not specified | No | No |
| Knudsen, A^15^ | 2021 | Norway | All people | "In accordance with the ICD-10" | Mar - May (2014-2018) | Mar - May 2020 | Those 3 months per year | Norwegian Cause of Death Registry | Eurostat | No | No |
| Bray, J^16^ | 2021 | Maryland | All people | Not specified | 2017/2018 and 1 Jan 20 - 4 Mar 20 | 5 Mar 2020 - 7 Jul 2020 | Those 7 months per year | Chief Medical Examiner | Population data was not provided | No | No |
| Leske, S^17^ | 2021 | Queensland, Australia | All people | Suspected suicides | Jan 2015-Jan 2020 | Feb 2020 - Aug 2020 | Pre x during pandemic periods | interim Queensland Suicide Register (iQSR) | Australian Bureau of Statistics | Yes | No |
| Mitchell, T^18^ | 2021 | Connecticut | All people | Not specified | 10 March - 20 May (2014-2019) | 10 March - 20 May 2020 | Those 82 days per year | Chief Medical Examiner | 2000 US standard population | No | No |
| Schleihauf, E^19^ | 2022 | Nova Scotia | All people | Confirmed suicide deaths | Jan 2011 - February 2020 | March 2020 - February 2021 | 12 month period | Medical Examiner Service | Population data was not provided | No | No |
| Barbic, D^20^ | 2021 | British Columbia | All people | Death resulting from self-inflicted injury, with the intent to cause death | Jan - Dec 2019 | Not specified | Monthly | BC Coroners Service | Government of British Columbia official website | Yes | Yes |
| Appleby, L^21^ | 2021 | 10 regions in England | All people | Suspected suicides | Jan-Oct 2016-2018 | Jan-Oct 2020 | Monthly (Jan to Oct) | Real time surveillance from Sustainability and Transformation Partnership | Office for National Statistics | No | No |
| Arya, V^22^ | 2022 | India | All people | Not specified | 2010-2019 | 2020 | Annually | National Crime Records Bureau | Ministry of Health and Family Welfare | Yes | No |
| McIntyre, R^23^ | 2021 | Canada | All people | ICD X60-X84, Y87.0) | Mar-Feb 2010-2020 | Mar 2020 - Feb 2021 | Annually | Statistics Canada | Statistics Canada | No | No |
| Stene-Larsen, K^24^ | 2022 | Norway | All people | ICD X60-X84, Y87.0) | Jan 2010 - Feb 2020 | Mar 2020 - Dec 2020 | Annually and Jan-Feb/Mar-May/Jun-Sep/Oct-Dec | Norwegian Cause of Death Registry | European Standard Population | Yes | Yes |
| Pirkis, J^25^ | 2021 | Multiple areas/countries | All people | Not specified | Until March 2020 | April 2020 onwards | Monthly | Multiple | Population data was not provided | No | No |
| Chen, Y^26^ | 2022 | Taiwan | All people | Not specified | 2017-2019 | 2020 | Mean 2017-2019 and 2020 annual data | Taiwan Ministry of Health and Welfare | Population data was not provided | No | No |
| Pérez, V^27^ | 2022 | Catalonia, Spain | >18 | Not specified | 2019 | 2020 | Annually and monthly | Catalonia Regional Police Department | Population data was not provided | No | No |
| Wollschläger, D^28^ | 2021 | Rhineland–Palatinate (Germany) and  Emilia–Romagna (Italy) | All people | ICD X60-X84 | 2011-2019 | 2020 | Annually | Mortality Registries | State Statistical Office of Rhineland-Palatinate and Italian Institute of Statistics | No | No |
| Ando, M^29^ | 2022 | Japan | All people | Not specified | 2019 | 2020 | Annually | Japanese Ministry of Health Labor and Welfare | Population data was not used | Yes | Yes |
| Yorsaeng, R^30^ | 2022 | Thailand | All people | Not specified | 2019 | 2020 | Annually | Self-harm surveillance system (Report 506S) of the Thai Department of Mental Health | Government Agency Statistics | No | No |
| Kim, A^31^ | 2021 | South Korea | All people | Not specified | Jan-Aug 2019 | Jan-Aug 2020 | Monthly (from Jan to Aug) | Korean Statistical Information Service | Population data was not used | Yes | No |
| Behera, C^32^ | 2021 | South and South-east districts in New Delhi | All people | Not specified | Mar 25th - Oct 31st 2019 | Mar 25th - Oct 31st 2020 | Those 220 days per year | All India Institute of Medical Sciences | Population data was not used | Yes | No |
| Nomura, S^33^ | 2021 | Japan | All people | Not specified | 2016-2019 | 2020 | Monthly | Japanese Ministry of Health Labor and Welfare | Population data was not used | Yes | No |
| Zheng, X^34^ | 2021 | Guangdong, China | All people | ICD X60-X84, Y87.0 | Jan-Jun 2019 | Jan-Jun 2020 | Annually | Chinese Center for Disease Control and Prevention (CDC) Cause of Death Reporting System | Population Basic Information System | Yes | Yes |

#

Online resource 5: the table identifies each study by the first author’s last name and shows the following information: year of publication, country/place where the study took place, population, definitions of suicide, definition of pre pandemic period, definition of during pandemic period, frequency of data reported, data source of cases and of the population and if the study included data about gender and age groups.

# Online Resource 6: Excluded studies from meta-analysis

General population:

Data from 21 out of the 34 included studies were used in the meta-analysis. The reason for exclusion of those 13 studies are shown by the table below.

| **Study Author** | **Reason for exclusion of the meta-analysis** |
| --- | --- |
| Matsumoto^5^, Tanaka^9^, Arya^22^ and Mitchell^18^ | Did not provide enough data for meta-analysis |
| Watanabe^8^ (Japan) | The number of suicide cases do not match with the data from other studies which has the same population (Inoue^53^, Fushimi^57^, Ando^65^, Nomura^69^) and were supposed to have been extracted from the same data source. Therefore, another study was selected to represent the population of Japan |
| Inoue^6^/Ando^29^/Fushimi^10^ (Japan) | The pre pandemic period of those three studies was the year 2019. Another study with the same population (Nomura^69^) presented data of a longer pre pandemic period (from 2017 to 2019), therefore, data from Nomura^69^ was selected to be analysed. All of those studies had the same quality appraisal results. |
| Sakamoto^1^ (Japan) | The pre and during pandemic periods are shorter than the ones in the Nomura^69^ study. |
| Pirkis^25^ (Japan) | Another study (Nomura^69^) had a longer “during pandemic” period and the same quality appraisal results. |
| Sánchez^11^ (Japan) | The study provides rates per age group but not the general population rate. |
| Pirkis^25^ (South Korea) | Another study (Kim^67^) had a longer during-pandemic period (both had the same quality appraisal results). |
| Pirkis^25^ (Queensland, Australia) | Another study (Leske^33^) had both pre and during pandemic periods longer than this study. Leske^33^ also had better quality appraisal results. |
| Qin^12^/Knudsen^15^ (Norway) | Another study (Stene-Larsen^63^) had longer pre and during pandemic periods. Even though Knudsen^60^ had better quality appraisal results than Stene-Larsen^63^, the second was preferred considering the longer pre and during pandemic periods and the good quality appraisal result. |
| Pirkis^25^ (British Columbia, Canada) | Another study (Barbic^62^) had longer pre and during pandemic periods and also better quality appraisal results. |
| Odd^3^ | Population is people under 19 years of age |

Gender:

Data from 9 out of the 34 included studies were used in the meta-analysis. The reason for exclusion of those 25 studies are shown by the table below.

| **Study Author** | **Reason for exclusion of the meta-analysis** |
| --- | --- |
| Acharya^2^, Deisenhammer^4^, Buciuta^7^, Tanaka^9^, Fushimi^10^, Sáchez^11^, Calati^14^, Knudsen^15^, Bray^47^, Mitchell^18^, Schleihauf^19^, Appleby^21^, McIntyre^23^, Pirkis^25^, Wollschlager^28^, Yorsaeng^30^ | Did not provide data by gender |
| Matsumoto^5^ (Japan) and Arya^22^ (India) | Provided gender data, but not sufficient for meta-analysis |
| Nomura^33^ (Japan) | Although the study has a longer pre pandemic period compared to the two selected studies representing Japan, the data between Nomura^69^ and Inoue^53^/Ando^65^ had disparities, therefore, the data shown by more than one study was chosen to compose the meta-analysis. All three studies had the same quality assessment results. |
| Watanabe^8^ (Japan) | The data between Watanabe^43^ and Inoue^53^/Ando^65^ had disparities, therefore, the data shown by more than one study was chosen to compose the meta-analysis. All three studies had the same quality assessment results. |
| Sakamoto^1^ (Japan) | The pre and during pandemic periods are shorter than the ones in the Inoue^53^/Ando^65^ studies. |
| Qin^12^ (Norway) | Another study (Stene-Larsen^63^) had longer pre and during pandemic periods. Both studies had the same quality assessment results. |
| Odd^3^ | Population is people under 19 years of age |
| Arya^22^ | Insufficient data (did not provide total number of suicide cases) |

# Online Resource 7: Forest plots of the pooled suicide rate for the pre pandemic period (A) and the during pandemic period (B) by WHO region

A
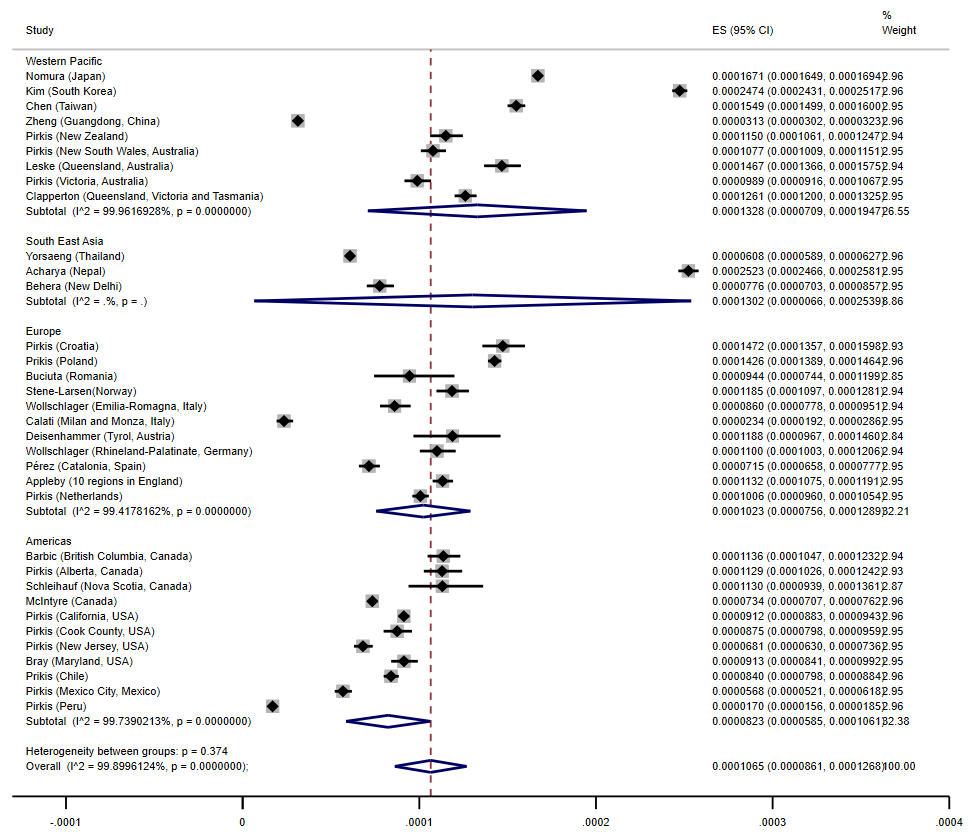


B


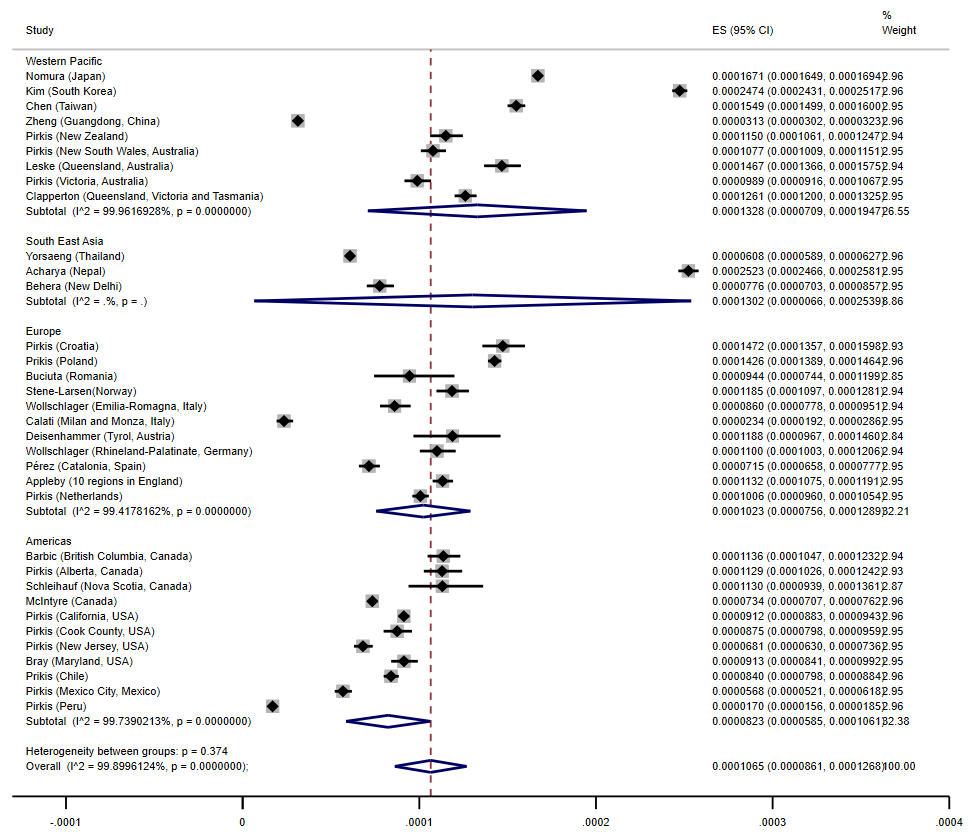


Online resource 7: on the left of the forest plots we find the studies (total of 34) divided by WHO regions and on the right we see the prevalence of suicide for each study.

# Online Resource 8: Forest plot of the pooled suicide rate for the pre and during pandemic periods among women (A) and men (B)

A
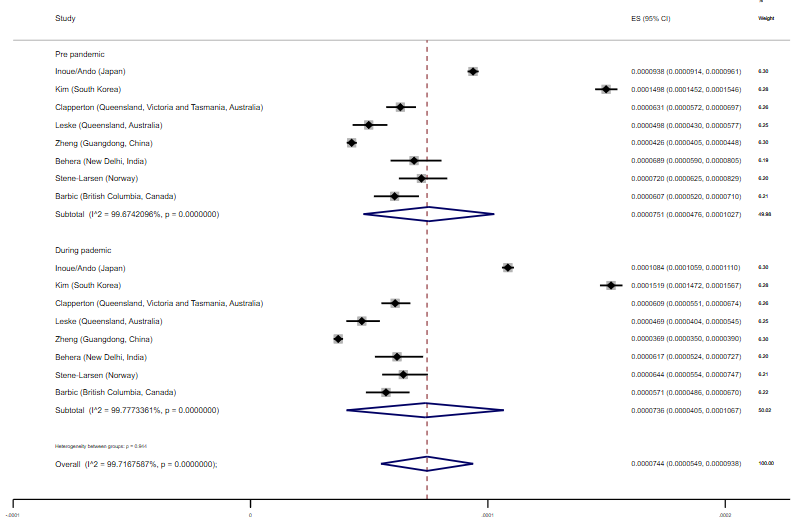


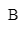

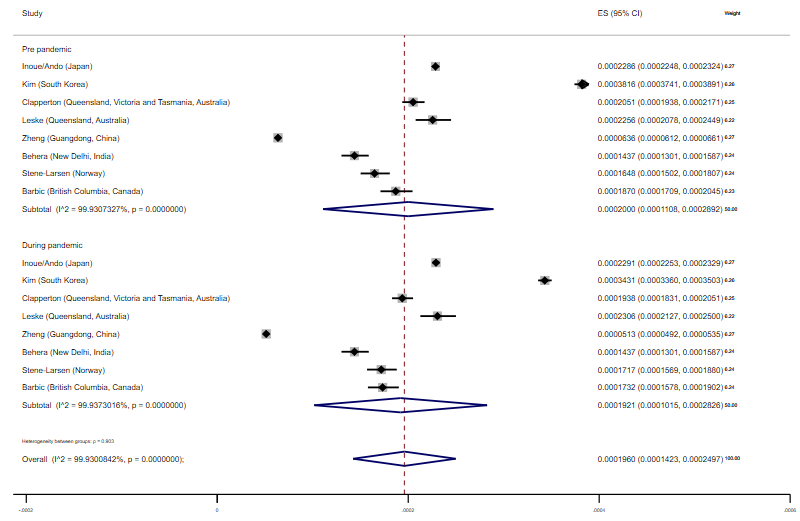


Online resource 8: on the left of the forest plots we find the studies (total of 8) divided between before and during the pandemic and on the right we see the prevalence of suicide for each study.

#

#

#

#

#

#

#

#

#

#

#

#

#

#

#

#

#

#

#

#

#

#

#

#

#

#

#

#

#

#

#

#

#

#

#

#

# Online Resource 9: Estimated suicide rates (95% CI)/100,000 by age groups*

|  | **Age group** | **Pre pandemic** | **During pandemic** |
| --- | --- | --- | --- |
| Japan (Ando^29^) | <20 | 3.18 (2.93-3.43) | 3.75 (3.48-4.02) |
|  | 20-29 | 16.66 (15.94-17.39) | 19.84 (19.05-20.63) |
|  | 30-39 | 17.77 (17.07-18.48) | 18.36 (17.64-19.08) |
|  | 40-49 | 18.68 (18.04-19.31) | 19.45 (18.80-20.10) |
|  | 50-59 | 20.6 (19.89-21.30) | 20.54 (19.83-21.24) |
|  | 60-69 | 18.51 (17.82-19.20) | 18.00 (17.32-18.68) |
|  | 70-79 | 17.95 (17.28-18.61) | 18.62 (17.94-19.29) |
|  | 80+ | 27.16 (26.19-28.14) | 19.98 (19.15-20.81) |
| British Columbia, Canada (Barbic^20^) | <20 | 2.27 (1.30-3.24) | 2.06 (1.14-2.98) |
|  | 20-29 | 17.57 (14.24-20.91) | 15.83 (12.66-19.00) |
|  | 30-39 | 11.8 (9.22-14.37) | 15.1 (12.19-18.01) |
|  | 40-49 | 16.99 (13.71-20.28) | 15.16 (12.06-18.27) |
|  | 50-59 | 16.78 (13.65-19.91) | 16.56 (13.45-19.67) |
|  | 60-69 | 15.86 (12.80-18.93) | 11.34 (8.75-13.93) |
|  | 70-79 | 12.57 (9.24-15.90) | 9.7 (6.78-12.63) |
|  | 80+ | 12.73 (8.16-17.30) | 13.35 (8.66-18.03) |
| Norway (Stene-Larsen^24^) | 0-24 | 6.57 (5.16-7.99) | 5.89 (4.54-7.23) |
|  | 15-24 | 12.39 (9.65-15.13) | 10.88 (8.32-13.45) |
|  | 25-44 | 15.71 (13.62-17.80) | 15.36 (13.29-17.43) |
|  | 45-64 | 16.45 (14.26-18.64) | 15.14 (13.04-17.24) |
|  | 65+ | 12.41 (10.08-14.73) | 14.47 (11.96-16.98) |
| Guangdong, China (Zheng^34^) | 10-14 | 0.63 (0.35-0.91) | 1.5 (1.06-1.94) |
|  | 15-59 | 3.81 (3.61-4.01) | 2.95 (2.78-3.12) |
|  | 60-69 | 8.4 (7.38-9.42) | 6.11 (5.33-6.89) |
|  | 70-79 | 7.36 (6.22-8.50) | 8.6 (7.31-9.89) |
|  | 80+ | 10.57 (8.59-12.55) | 8.17 (6.40-9.94) |
| Queensland, Victoria and Tamania, Australia (Clapperton^13^) | <25 | 5.73 (4.96-6.50) | 6.54 (5.72-7.37) |
|  | 25-64 | 18.62 (17.51-19.73) | 17.6 (16.52-18.68) |
|  | 65+ | 12.33 (10.73-13.93) | 13.00 (11.35-14.64) |
| England (Odd^3^) | <19 | 0.85 (0.64 - 1.07) | 0.78 (0.58 - 0.99) |

*All rates were estimated by the author using suicide data extracted from the studies, except for the values of the study published by Ando^29^ and Odd^3^, which were directly extracted from the study.

Online resource 9: the table shows suicide rates by age groups of 6 studies/locations (Japan, British Columbia, Norway, Guangdong, Australia and England. The age groups are different between the studies.

# Online Resource 10: Estimated suicide rates (95% CI)/100,000 by ethnicity

| **Area** | **Ethnicity** | **Pre pandemic** | **During pandemic** |
| --- | --- | --- | --- |
| **Maryland [16] (USA)** | Black | 4.94 (3.85-6.03) | 5.88 (4.69-7.07) |
|  | White | 13.65 (12.37-14.94) | 9.47 (8.41-10.54) |
|  | Hispanic | 2.11 (0.76-3.46) | 2.96 (1.36-4.56) |
|  | Asian | 3.12 (1.12-5.12) | 4.99 (2.47-7.52) |
|  | Other | 2.9 (0.50-5.29) | 2.9 (0.5-5.29) |
| **Connecticut [18](USA)** | White | 14.07 (11.62-15.53) | 11.01 (9.73-12.30) |
|  | Non-white | 3.64 (2.61-4.67) | 6.4 (5.03-7.77) |

Online resource 10: the table shows pre and during pandemic suicide rates among different ethnicities for 2 studies. The study in Maryland divided ethnicity by black, white, hispanic, asian and other, while the study in Connecticut categorized as white and non-white only.

# Online Resource 11: Estimated Suicide rates (95% CI)/100,000 by socio-demographic index (SDI)

| **Area** | **SDI** | **Pre pandemic** | **During pandemic** |
| --- | --- | --- | --- |
| **India^22^** | Low SDI | 6.06 (6.00-6.12) | 6.77 (6.71-6.84) |
|  | Middle SDI | 13.72 (13.61-13.84) | 14.34 (14.22-14.46) |
|  | High SDI | 15.6 (15.46-15.74) | 17.38 (17.23-17.52) |

Online resource 11: the table shows pre and during pandemic suicide rates according to socio-demographic index (categorized as low, middle and high) in India. The highest suicide rate is seen among high SDI and there was a small increase between the periods in all of the categories (steepest in the high SDI group).

# Online Resource 12: Funnel plot of studies investigating suicide rates in the pre and during Covid-19 pandemic periods

#
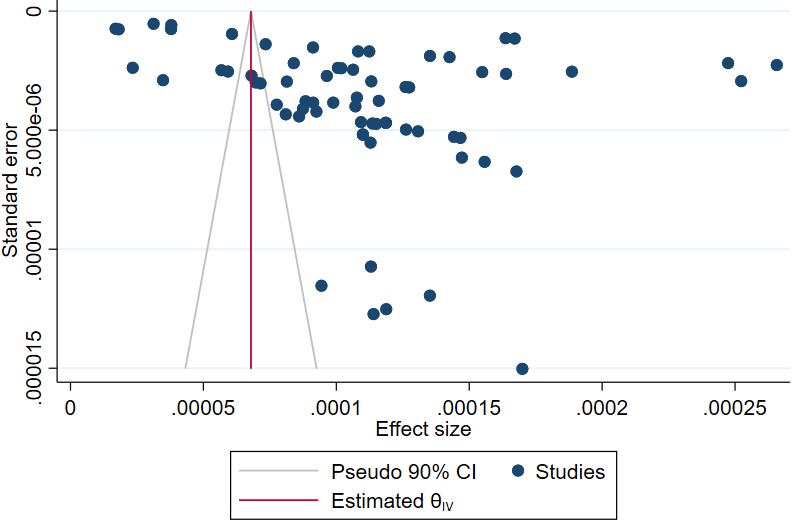


#

#

Online resource 12: the figure shows a triangle with the base at the bottom. There are scattered dots located especially at the top of the image and outside the triangle.

# Online Resource 13: GRADE Evidence Profile of all Observational Studies included in the Systematic Review

| Outcome Measure | Number of Studies | Risk of bias | Imprecision | Inconsistency | Indirectness | Publication Bias | Certainty of Evidence |
| --- | --- | --- | --- | --- | --- | --- | --- |
| Suicide rate | 34 | Serious risk  (14 studies were unclear about data sources) | No serious imprecision | Very serious inconsistency  (Large variation in size of effect, large I2 value and chi square test) | No serious indirectness | Detected  (funnel plot) | Very low |

Online resource 13: the table informs us that the outcome measure is suicide rate; the number of studies is 34; the risk of bias is serious (because 14 studies were unclear about data sources); there was no serious imprecision, inconsistency was very serious (due to large variation is size of effect, large I2 value and chi square test), no serious indirectness, presence of publication bias detected through the funnel plot and the certainty of evidence in considered very low.

#

#

#

#

#

#

#

#

#

# Online Resource 14: Results of the meta-regression between WHO regions

The table presents the results of a meta-regression analysis investigating the influence of WHO regions on suicide rates during the COVID-19 pandemic.

#

| **WHO Region** | **Number of studies** | **Coefficient** | **Std. Error** | **95% CI** | **P-value** |
| --- | --- | --- | --- | --- | --- |
| **Western Pacific** | 9 | 0 (reference) | | | |
| **South East Asia** | 3 | 8.34e-06 | 0.0000208 | -0.000034 | 0.691 |
| **Europe** | 11 | 0.0000123 | 0.0000317 | -0.000052 | 0.701 |
| **Americas** | 11 | 0.00004 | 0.0000219 | -4.66E-06 | 0.077 |

#

# References

1. Sakamoto H, Ishikane M, Ghaznavi C, Ueda P. Assessment of Suicide in Japan During the COVID-19 Pandemic vs Previous Years. JAMA Netw Open. 2021;4(2):e2037378. Published 2021 Feb 1. doi:10.1001/jamanetworkopen.2020.37378

2. Acharya B, Subedi K, Acharya P, Ghimire S. Association between COVID-19 pandemic and the suicide rates in Nepal. Leong C, ed. PLoS ONE. 2022;17(1):e0262958. doi:10.1371/journal.pone.0262958

3. Odd D, Williams T, Appleby L, Gunnell D, Luyt K. Child suicide rates during the COVID-19 pandemic in England. *J Affect Disord Rep*. 2021;6:100273. doi:10.1016/j.jadr.2021.100273

4. Deisenhammer EA, Kemmler G. Decreased suicide numbers during the first 6 months of the COVID-19 pandemic. *Psychiatry Res*. 2021;295:113623. doi:10.1016/j.psychres.2020.113623

5. Matsumoto R, Motomura E, Fukuyama K, Shiroyama T, Okada M. Determining What Changed Japanese Suicide Mortality in 2020 Using Governmental Database. *J Clin Med*. 2021;10(21):5199. Published 2021 Nov 7. doi:10.3390/jcm10215199

6. Inoue K, Fukunaga T. Discussion of the features of suicide trends in 1994-1995 and 2019-2020: The need for different contents in suicide prevention measures for both periods in Japan from the viewpoint of the present. *Leg Med (Tokyo)*. 2022;58:102083. doi:10.1016/j.legalmed.2022.102083

7. Vuscan ME, Buciuta A, Vica ML, et al. Impact of the COVID-19 Pandemic on the Suicidal Behavior in Romania [published online ahead of print, 2022 Jan 18]. *Arch Suicide Res*. 2022;1-11. doi:10.1080/13811118.2021.2022052

8. Watanabe M, Tanaka H. Increased suicide mortality in Japan during the COVID-19 pandemic in 2020. *Psychiatry Res*. 2022;309:114422. doi:10.1016/j.psychres.2022.114422

9. Tanaka T, Okamoto S. Increase in suicide following an initial decline during the COVID-19 pandemic in Japan. Nat Hum Behav. 2021;5(2):229-238. doi:10.1038/s41562-020-01042-z

10. Fushimi M. Is the COVID-19 pandemic a factor that led to completed suicides among suicide attempters?. *Psychiatry Res*. 2022;312:114569. doi:10.1016/j.psychres.2022.114569

11.Ruiz Sánchez G. Monthly suicide rates during the COVID-19 pandemic: Evidence from Japan. *Econ Lett*. 2021;207:110014. doi:10.1016/j.econlet.2021.110014

12. Qin P, Mehlum L. National observation of death by suicide in the first 3 months under COVID‐19 pandemic. Acta Psychiatr Scand. 2021;143(1):92-93. doi:10.1111/acps.13246

13. Clapperton A, Spittal MJ, Dwyer J, et al. Patterns of Suicide in the Context of COVID-19: Evidence From Three Australian States. *Front Psychiatry*. 2021;12:797601. Published 2021 Nov 30. doi:10.3389/fpsyt.2021.797601

14. Calati R, Gentile G, Fornaro M, Tambuzzi S, Zoja R. Preliminary suicide trends during the COVID-19 pandemic in Milan, Italy. *J Psychiatr Res*. 2021;143:21-22. doi:10.1016/j.jpsychires.2021.08.029

15. Knudsen AKS, Stene-Larsen K, Gustavson K, et al. Prevalence of mental disorders, suicidal ideation and suicides in the general population before and during the COVID-19 pandemic in Norway: A population-based repeated cross-sectional analysis. Lancet Reg Health Eur. 2021;4:100071. Published 2021 Feb 27. doi:10.1016/j.lanepe.2021.100071

16. Bray MJC, Daneshvari NO, Radhakrishnan I, et al. Racial Differences in Statewide Suicide Mortality Trends in Maryland During the Coronavirus Disease 2019 (COVID-19) Pandemic. JAMA Psychiatry. 2021;78(4):444. doi:10.1001/jamapsychiatry.2020.3938

17. Leske S, Kõlves K, Crompton D, Arensman E, de Leo D. Real-time suicide mortality data from police reports in Queensland, Australia, during the COVID-19 pandemic: an interrupted time-series analysis. The Lancet Psychiatry. 2021;8(1):58-63. doi:10.1016/s2215-0366(20)30435-1

18. Mitchell TO, Li L. State-Level Data on Suicide Mortality During COVID-19 Quarantine: Early Evidence of a Disproportionate Impact on Racial Minorities. Psychiatry Research. 2021;295:113629. doi:10.1016/j.psychres.2020.113629

19. Schleihauf E, Bowes MJ. Suicide and drug toxicity mortality in the first year of the COVID-19 pandemic: use of medical examiner data for public health in Nova Scotia. Mortalité par suicide et par intoxication aux drogues ou aux médicaments dans la première année de la pandémie de COVID-19 : utilisation des données des médecins légistes à des fins de santé publique en Nouvelle-Écosse. *Health Promot Chronic Dis Prev Can*. 2022;42(2):60-67. doi:10.24095/hpcdp.42.2.02

20. Barbic D, Scheuermeyer FX, Barbic SP, Honer WG. Suicide Deaths in British Columbia during the First Wave of the COVID-19 Pandemic. Can J Psychiatry. 2021;66(9):830-831. doi:10.1177/07067437211018398

21. Appleby L, Richards N, Ibrahim S, Turnbull P, Rodway C, Kapur N. Suicide in England in the COVID-19 pandemic: Early observational data from real time surveillance. The Lancet Regional Health - Europe. 2021;4:100110. doi:10.1016/j.lanepe.2021.100110

22. Arya V, Page A, Spittal MJ, et al. Suicide in India during the first year of the COVID-19 pandemic. Journal of Affective Disorders. 2022;307:215-220. doi:10.1016/j.jad.2022.03.06

23. McIntyre RS, Lui LM, Rosenblat JD, et al. Suicide reduction in Canada during the COVID-19 pandemic: lessons informing national prevention strategies for suicide reduction. J R Soc Med. 2021;114(10):473-479. doi:10.1177/01410768211043186

24. Stene-Larsen K, Raknes G, Engdahl B, et al. Suicide trends in Norway during the first year of the Covid-19 pandemic. A register-based cohort study [published online ahead of print, 2022 Apr 19]. *Eur Psychiatry*. 2022;65(1):1-24. doi:10.1192/j.eurpsy.2022.17

25. Pirkis J, John A, Shin S, et al. Suicide trends in the early months of the COVID-19 pandemic: an interrupted time-series analysis of preliminary data from 21 countries. The Lancet Psychiatry. 2021;8(7):579-588. doi:10.1016/s2215-0366(21)00091-2

26. Chen YY, Yang CT, Pinkney E, Yip PSF. Suicide trends varied by age-subgroups during the COVID-19 pandemic in 2020 in Taiwan. *J Formos Med Assoc*. 2022;121(6):1174-1177. doi:10.1016/j.jfma.2021.09.021

27. Pérez V, Elices M, Vilagut G, et al. Suicide-related thoughts and behavior and suicide death trends during the COVID-19 in the general population of Catalonia, Spain [published correction appears in Eur Neuropsychopharmacol. 2023 Jan;66:66]. *Eur Neuropsychopharmacol*. 2022;56:4-12. doi:10.1016/j.euroneuro.2021.11.006

28. Wollschläger D, Schmidtmann I, Blettner M, et al. Suicides During the COVID-19 Pandemic 2020 Compared to the Years 2011-2019 in Rhineland-Palatinate (Germany) and Emilia-Romagna (Italy). *Dtsch Arztebl Int*. 2021;118(47):814-815. doi:10.3238/arztebl.m2021.0365

29. Ando M, Furuichi M. The association of COVID-19 employment shocks with suicide and safety net use: An early-stage investigation. *PLoS One*. 2022;17(3):e0264829. Published 2022 Mar 24. doi:10.1371/journal.pone.0264829

30. Yorsaeng R, Suntronwong N, Thongpan I, et al. The impact of COVID-19 and control measures on public health in Thailand, 2020. *PeerJ*. 2022;10:e12960. Published 2022 Feb 16. doi:10.7717/peerj.12960

31. Kim AM. The short-term impact of the COVID-19 outbreak on suicides in Korea. *Psychiatry Res*. 2021;295:113632. doi:10.1016/j.psychres.2020.113632

32. Behera C, Gupta SK, Singh S, Balhara YPS. Trends in deaths attributable to suicide during COVID-19 pandemic and its association with alcohol use and mental disorders: Findings from autopsies conducted in two districts of India. *Asian J Psychiatr*. 2021;58:102597. doi:10.1016/j.ajp.2021.102597

33. Nomura S, Kawashima T, Yoneoka D, et al. Trends in suicide in Japan by gender during the COVID-19 pandemic, up to September 2020. *Psychiatry Res*. 2021;295:113622. doi:10.1016/j.psychres.2020.113622

34. Zheng XY, Tang SL, Ma SL, et al. Trends of injury mortality during the COVID-19 period in Guangdong, China: a population-based retrospective analysis. BMJ Open. 2021;11(6):e045317. Published 2021 Jun 2. doi:10.1136/bmjopen-2020-045317
